# Development of an individualized risk calculator for poor functioning in young people victimized during childhood: A longitudinal cohort study

**DOI:** 10.1016/j.chiabu.2019.104188

**Published:** 2019-12

**Authors:** Rachel M. Latham, Alan J. Meehan, Louise Arseneault, Daniel Stahl, Andrea Danese, Helen L. Fisher

**Affiliations:** a King’s College London, Social, Genetic & Developmental Psychiatry Centre, Institute of Psychiatry, Psychology & Neuroscience, London, UK; b King’s College London, Department of Biostatistics, Institute of Psychiatry, Psychology & Neuroscience, London, UK; c King’s College London, Department of Child & Adolescent Psychiatry, Institute of Psychiatry, Psychology & Neuroscience, London, UK; d National and Specialist CAMHS Trauma, Anxiety, and Depression Clinic, South London and Maudsley NHS Foundation Trust, London, UK

**Keywords:** Childhood victimization, Functioning, Maltreatment, Prediction modelling, Resilience, Risk

## Abstract

**Background:**

Childhood victimization elevates the average risk of developing functional impairment in adulthood. However, not all victimized children demonstrate poor outcomes. Although research has described factors that confer vulnerability or resilience, it is unknown if this knowledge can be translated to accurately identify the most vulnerable victimized children.

**Objective:**

To build and internally validate a risk calculator to identify those victimized children who are most at risk of functional impairment at age 18 years.

**Participants:**

We utilized data from the Environmental Risk (E-Risk) Longitudinal Twin Study, a nationally-representative birth cohort of 2232 UK children born in 1994–95.

**Methods:**

Victimization exposure was assessed repeatedly between ages 5 and 12 years along with a range of individual-, family- and community-level predictors. Functional outcomes were assessed at age 18 years. We developed and evaluated a prediction model for psychosocial disadvantage and economic disadvantage using the Least Absolute Shrinkage and Selection Operator (LASSO) regularized regression with nested 10-fold cross-validation.

**Results:**

The model predicting psychosocial disadvantage following childhood victimization retained 12 of 22 predictors, had an area under the curve (AUC) of 0.65, and was well-calibrated within the range of 40–70% predicted risk. The model predicting economic disadvantage retained 10 of 22 predictors, achieved excellent discrimination (AUC = 0.80), and a high degree of calibration.

**Conclusions:**

Prediction modelling techniques can be applied to estimate individual risk for poor functional outcomes in young adulthood following childhood victimization. Such risk prediction tools could potentially assist practitioners to target interventions, which is particularly useful in a context of scarce resources.

## 1 Introduction

Child victimization – including abuse, neglect, bullying and exposure to domestic violence – is a severe infringement of human rights that impacts a substantial proportion of individuals worldwide (Gilbert et al., 2009; UNICEF, 2014), including nearly 1-in-5 British children (Radford, Corral, Bradley, & Fisher, 2013). Research into the long-term consequences of early victimization suggests that exposed children are at elevated risk for a range of adverse outcomes, including functional impairment. For example, childhood bullying and maltreatment have both been associated with poorer occupational outcomes in adulthood including elevated risk for being ‘Not in Employment, Education, or Training’ (NEET) (Jaffee et al., 2018) or unemployed (Brimblecombe et al., 2018), lower levels of education, lower earnings, and less likelihood of being in skilled employment (Currie & Widom, 2010). Moreover, experiences of childhood abuse have been shown to increase the risk of criminal offending (Malvaso, Delfabbro, & Day, 2018), teenage parenthood (Herrenkohl, Herrenkohl, Egolf, & Russo, 1998), and to be linked with lower self-esteem, life satisfaction (Fergusson, McLeod, & Horwood, 2013), and poorer sleep quality (Anda et al., 2006) in adulthood. Such outcomes may have negative and long-lasting implications for individuals’ wellbeing and life opportunities. They also entail significant costs for public services and the community through lost employment productivity, unemployment benefits, criminality, and incarceration.

Although victimized children have, on average, poorer functional outcomes compared to their non-victimised peers, these group differences fail to capture the remarkable resilience that many victimized children display. Indeed, there is significant within-group variation in functioning with some individuals demonstrating positive outcomes despite their victimization experiences (Cicchetti, 2013; Klika & Herrenkohl, 2013). This highlights the importance of better understanding the determinants of resilience and vulnerability following childhood victimization to inform preventative interventions and enable resources to be targeted more effectively.

Accumulating research has described factors across individual, family, and community domains that are associated with poor functioning following exposure to victimization (Afifi & MacMillan, 2011; Meng, Fleury, Xiang, Li, & D’Arcy, 2018). However, it is unclear whether this theoretical knowledge can be translated to enable accurate identification of those victimized children who are most vulnerable. First, many studies in this area have used cross-sectional or only short-term longitudinal designs (Cicchetti, 2013). Therefore, any significant associations found provide little information about the direction of associations between childhood predictors and young people’s outcomes. Second, studies have often tested associations between only one predictor (or just a few predictors) and outcomes (Meng et al., 2018) rather than testing a comprehensive range of individual, family, and community predictors together. Such multivariable models are crucial to understand the co-occurring influences on a victimized child’s later functioning and the relative contribution of each factor. Third, existing studies identify factors that increase or decrease the average risk of functioning among victimized children, but none have tested whether these factors can accurately predict whether a particular victimized child will develop poor functioning or not. For example, the presence of social support has been shown to reduce the average risk of poor functioning among victimized children (Hyman & Williams, 2001). However, because some victimized children with social support develop poor functioning but others do not, it is unclear whether social support can be used to make individualized predictions of risk – particularly as existing models have not been assessed with new or unseen cases. Therefore, existing knowledge is of limited use to practitioners who seek insight into a specific victimized child’s level of vulnerability.

The current study addresses these three limitations. First, we use data from a nationally-representative 18-year longitudinal cohort of UK children. This prospective longitudinal design – with young people’s functioning measured after victimization exposure and vulnerability and resilience factors – clarifies the direction of associations, thereby addressing gaps in the literature from cross-sectional and short-term longitudinal studies. Second, the current sample provides a comprehensive assessment of victimization, individual, family, and community factors, and functional outcomes enabling the investigation of multivariable models of functioning following childhood victimization. Third, we employ prediction modelling techniques to test whether identified vulnerability and resilience factors can be used to accurately predict which victimized children are most vulnerable to poor functioning. Here, we draw on a recent systematic literature review (Meng et al., 2018) to develop a multivariable prediction model that calculates an individual’s personal risk for developing poor functional outcomes after childhood victimization. We adopt this approach to the selection of predictor variables because it is recommended as best practice for developing prediction models (Fusar-Poli, Hijazi, Stahl, & Steyerberg, 2018). We assess the model’s predictive accuracy with new cases using cross-validation methods to estimate internal validity (Steyerberg, 2009). Risk prediction tools such as this are widely used within medical practice (e.g. the Framingham Risk Score for cardiovascular disease; D’Agostino et al., 2008). However, their use in psychology and social work remains relatively novel despite their potential to assist practitioners’ decision-making.

## 2 Methods

### 2.1 Participants

Participants were members of the Environmental Risk (E-Risk) Longitudinal Twin Study, which tracks the development of a nationally-representative birth cohort of 2232 British twin children. The sample was drawn from a larger birth register of twins born in England and Wales in 1994–1995 (Trouton, Spinath, & Plomin, 2002). Full details about the sample are reported elsewhere (Moffitt & E-Risk Study Team, 2002). Briefly, the E-Risk sample was constructed in 1999–2000 when 1116 families (93% of those eligible) with same-sex 5-year-old twins participated in home-visit assessments. This sample comprised 56% monozygotic (MZ) and 44% dizygotic (DZ) twin pairs; sex was evenly distributed within zygosity (49% male). Families were recruited to represent the UK population of families with newborns in the 1990s, on the basis of residential location throughout England and Wales and mother’s age. Teenaged mothers with twins were over-selected to replace high-risk families who were selectively lost to the register through non-response. Older mothers having twins via assisted reproduction were under-selected to avoid an excess of well-educated older mothers.

Follow-up home-visits were conducted when the children were aged 7, 10, 12 and 18 years (participation rates were 98%, 96%, 96%, and 93%, respectively). Home-visits at ages 5, 7, 10, and 12 years included assessments with participants as well as their mother (or primary caregiver); the home-visit at age 18 years included interviews only with the participants. Each participant in a twin pair was assessed by a different interviewer. There were 2066 E-Risk participants who were assessed at age 18 years. The average age of the participants at the time of the assessment was 18.4 years (*SD* = 0.36); all interviews were conducted after the 18th birthday. There were no differences between those who did and did not take part at age 18 years in terms of socioeconomic status (SES) assessed when the cohort was initially defined (χ^2^ = 0.86, *p* = 0.65), age-5 IQ scores (*t* = 0.98, *p* = 0.33), age-5 behavioral (*t* = 0.40, *p* = 0.69) or emotional (*t* = 0.41, *p* = 0.68) problems, or childhood poly-victimization (*z* = 0.51, *p* = 0.61). E-Risk families are representative of UK households across the spectrum of neighborhood-level deprivation: 25.6% of E-Risk families live in “wealthy achiever” neighborhoods compared to 25.3% of households nation-wide; 5.3% vs 11.6% live in “urban prosperity” neighborhoods; 29.6% vs 26.9% live in “comfortably off” neighborhoods; 13.4% vs 13.9% live in “moderate means” neighborhoods; and 26.1% vs 20.7% live in “hard-pressed” neighborhoods (CACI Information Services, 2006; Caspi, Taylor, Moffitt, & Plomin, 2000). E-Risk underrepresents urban prosperity neighborhoods because such households are likely to be childless.

The Joint South London and Maudsley and the Institute of Psychiatry Research Ethics Committee approved each phase of the study. Parents gave informed consent and twins gave assent between 5–12 years and then informed consent at age 18 years.

### 2.2 Measures

#### 2.2.1 Childhood victimization

Lifetime exposure to several types of victimization was assessed repeatedly when E-Risk children were 5, 7, 10, and 12 years of age, and comprehensive dossiers have been compiled for each child with cumulative information about exposure between birth and age 12 years to domestic violence between the mother and her partner; frequent bullying by peers; physical abuse by an adult; sexual abuse; emotional abuse/neglect; and physical neglect. The dossiers comprised reports from caregivers of victimization using questions based on the MultiSite Child Development Project (Dodge, Bates, & Pettit, 1990; Lansford et al., 2002), recorded narratives of the caregiver interviews, recorded debriefings with research workers who had coded any indication of abuse and neglect at any of the successive home visits using the Home Observation for Measurement of the Environment (HOME) (Bradley & Caldwell, 1977), and information from clinicians whenever the study team made a child-protection referral. The dossiers were reviewed by two independent researchers and rated for the presence and severity (none/mild/severe) of each type of victimization. Inter-rater agreement between the coders exceeded 85% among the positive cases, and discrepantly coded cases were resolved by consensus review. In the present study, prospectively-reported victimization was dichotomized to represent none/mild (0) versus severe (1) victimization. Those classified as having experienced any type of severe childhood victimization (*n* = 591; 26.5% of sample) were selected for subsequent analyses. Full details have been reported previously (Danese et al., 2016; Fisher et al., 2015) and in the Supplementary Materials.

#### 2.2.2 Age-18 functional outcomes

We assessed nine functional outcomes at age 18 years. Cautions and convictions were assessed through the UK Police National Computer (PNC) record searches; all other outcomes were assessed at the age-18 interview. Not in Education, Employment or Training (NEET) status, parenthood, and cautions and convictions were naturally dichotomous; all other variables were dichotomized. For variables with no predetermined cut-off (social isolation, low life satisfaction, loneliness, and low sleep quality) we defined poor functioning as being among the 20% worst scoring participants for an outcome (as previously used in this cohort: Wertz et al., 2018). Functional outcome measures are summarized in Table 1 with detailed descriptions in Supplementary materials

**Table 1:** Summary of Age-18 Functional Outcome Measures.

Outcome	Description
Low educational achievement^a^	Did not obtain any school-leaving qualification(s) or only at low grades (GCSE grades D-G) and did not obtain any A-levels.
NEET status	Participant is classified as NEET if not studying, nor working in paid employment, nor pursuing a vocational qualification or apprenticeship.
Parenthood	Any past live birth or current pregnancy (girls) or having caused a pregnancy that resulted in a live birth (boys).
Cautions^b^ and convictions	Official record of any UK caution or convictions, beginning at age 10 years, the age of criminal responsibility.
Adolescent poly-victimization	Experience of two or more types of victimization between ages 12 and 18 years. Assessed using the JVQ, adapted as a clinical interview covering 7 categories of victimization: crime victimization, peer/sibling victimization, cyber-victimization, sexual victimization, family violence, maltreatment, and neglect.
Social isolation	High score (among the top 20% of participants) on the Multidimensional Scale of Perceived Social Support. Reverse-coded to assess social isolation.
Low life satisfaction	High score (among the top 20% of participants) on the Satisfaction with Life Scale reverse-coded to assess low life satisfaction.
Loneliness	High score (among the top 20% of participants) on the UCLA Loneliness Scale.
Low sleep quality	High score (among the top 20% of participants) on the Sleep Quality Index.

*Note*. GCSE = General Certificate of Secondary Education; NEET = Not in Employment, Education or Training; JVQ = Juvenile Victimization Questionnaire.

a In the United Kingdom, students are eligible to leave school upon completion of the GCSE examination at age 16 years. Some students remain in school for an additional 2 years to complete Advanced level (A-level or equivalent) qualifications, which are required for university entrance. Participants with poor educational qualifications were those who did not obtain their A-level qualifications or scored a low grade (D–G) on their GCSE examinations.

b A caution (warning) is a formal alternative to prosecution when a minor crime is committed, and the individual admits the offence. See Supplementary materials for full details of measures and references.

For data reduction, we performed exploratory factor analysis (principal factors with varimax rotation). Because our variables are dichotomous, this was performed on the polychoric correlation matrix. Results suggested a two-factor solution based on eigenvalues greater than 1 (Supplementary Table S1). We then ran a confirmatory factor analysis (maximum-likelihood factor method with orthogonal rotation) specifying two factors (Supplementary Table S2). The first factor – conceptualized as ‘psychosocial disadvantage’ – comprised adolescent poly-victimization, social isolation, low life satisfaction, loneliness, and low sleep quality. The second – conceptualized as ‘economic disadvantage’ – comprised low educational achievement, NEET status, parenthood, and criminal cautions and convictions. Accordingly, we created two age-18 outcome variables capturing whether individuals had any: (i) psychosocial disadvantage (1 = yes; 0 = no), or (ii) economic disadvantage (1 = yes; 0 = no).

#### 2.2.3 Childhood predictors

Individual-, family-, and community-level predictors were assessed between ages 5 and 12 years. We utilized a recent systematic review of multi-level predictors of maltreatment outcomes (Meng et al., 2018) and mapped the variables identified to those measured in the E-Risk Study (Supplementary Table S3). This approach is preferable to selecting predictors based on a statistically significant bivariate association with the outcome in the target sample, as it avoids the circular logic of testing the prediction of variables that are known to be associated with the outcome within that sample (Fusar-Poli et al., 2018). From this, we identified 22 predictors, summarized in Table 2 with detailed descriptions provided in the Supplementary Materials. This number of predictors ensured there was a minimum of 10 individuals with each outcome per predictor (314 victimized children had psychosocial disadvantage and 235 had economic disadvantage) to mitigate potential model instability due to over-fitting (Pavlou et al., 2015).

**Table 2:** Summary of Childhood Predictor Variables.

Measure	Age	Informant	Description	Score range
**Individual**				
Sex	5	Mother	1 = Male; 2 = Female	
IQ	12	Child	Pro-rated WISC-R score (Matrix Reasoning and Information subtests)	
Openness to experience	12	Researcher	Sum of 5 BFI items	0–10
Conscientiousness			Sum of 6 BFI items	0–12
Extraversion			Sum of 6 BFI items	
Agreeableness			Sum of 5 BFI items	0–10
Neuroticism			Sum of 5 BFI items	0–10
ADHD symptoms	12	Mother/teacher	Count of 18 DSM-IV inattentive and impulsive-hyperactive symptoms from CBCL (mothers) and TRF items (teachers), averaged across raters	0–18
Conduct disorder symptoms	12	Mother/teacher	Count of 14 DSM-IV criteria from CBCL (mothers) and TRF items (teachers), averaged across raters	0-14
Anxiety	12	Child	Symptom score from 10 MASC items	0–20
Depression	12	Child	Symptom score from 27 CDI items	0–54
Self-harm/suicide attempt	12	Mother	Any deliberate self-harm or attempted suicide in previous six months	
Psychotic symptoms	12	Child	Presence of at least one definite psychotic symptom	
**Family**				
Maternal warmth	5;10	Mother	Warmth, enthusiasm, interest in, enjoyment of child during FMSS, summed across time-points	0–10
Sibling warmth	7;10	Mother	6 items each, summed across time-points	0–24
Adult involvement	12	Child	Sum of 13 items assessing presence of a supportive adult	0–26
Family history of psychopathology	12	Mother	Proportion (0.0–1.0) of family members (parents, grandparents, aunts/uncles) with history of psychiatric disorder	
Biological parents in household	10	Mother	Number of biological parents in household up to age 10 (0 = both always absent; 1 = one absent at some point; 2 = both always present)	
Socioeconomic status	5	Mother	Tertiles derived from standardized composite of parental income, education, and occupation	
**Community**				
Neighborhood crime victimization	5	Mother	Sum of 3 items assessing family’s experience of violent crime, burglary, or theft in local area	0–6
Social cohesion	5	Mother	Sum of 5 items (neighbors close-knit, share values, trust each other, etc.)	0–10
Status among peers	12	Child	Self-selected position within 5 peer status ‘circles’	0–4

*Note.* Child’s age given in years. ADHD = attention-deficit/hyperactivity disorder; BFI = Big Five Inventory; CBCL = Child Behavior Checklist; CDI = Children’s Depression Inventory; DSM-IV = Diagnostic and Statistical Manual of Mental Disorders, Fourth Edition; IQ = intelligence; FMSS = Five-Minute Speech Sample; MASC = Multidimensional Anxiety Scale for Children; TRF = Teacher’s Report Form; WISC-R = Wechsler Intelligence Scale for Children-Revised. See Supplementary Materials for full details of measures and references.

### 2.3 Statistical analysis

Analyses were conducted using STATA 15.0 (Stata-Corp) and R version 3.5.1 (R Core Team, 2018). First, we tested whether the prevalence of psychosocial and economic disadvantage at age 18 years differed significantly between victimized and non-victimized participants using logistic regression, correcting for familial clustering using the “CLUSTER” command.

Second, we developed and internally validated prediction models for the two age-18 outcomes using regularized logistic regression with the *glmnet* R package (Friedman, Hastie, & Tibshirani, 2010). Of the 591 participants exposed to childhood victimization, 506 (85.6%) had complete data for all predictors and outcome for inclusion in the model predicting psychosocial disadvantage, and 503 (85.1%) had complete data for inclusion in the model predicting economic disadvantage. Missing data was predominantly due to participants not completing the age-18 assessment. As the vast majority of victimized participants had full data available, only complete cases were utilized for the model development and internal validation analyses. Complete data was not predicted by family SES (high SES compared to low SES: OR = 1.21, *p* = 0.656), or age-18 functional outcomes (psychosocial disadvantage: OR = 0.60, *p* = 0.194; economic disadvantage: OR = 0.71 *p* = 0.296).

A full description of our analyses is provided in the Supplementary Materials. Briefly, we conducted regularized regression using the Least Absolute Shrinkage and Selection Operator (LASSO) to identify subsets of predictors that maximized the prediction accuracy for the outcome variables in unseen cases. The LASSO shrinks coefficients towards zero thereby reducing the variance of these estimates (see Supplementary Fig. S2). Predictors with a regression coefficient shrunken to zero are excluded from the model enabling parsimonious solutions. The degree of shrinkage is determined by a tuning parameter, lambda (λ). Cross-validation was used to identify the optimal lambda for each model (Tibshirani, 1996). To obtain an estimate of prediction accuracy in new cases from the same underlying population, we internally validated each model using nested 10-fold cross-validation (Hastie, Tibshirani, & Friedman, 2009) (see Supplementary Fig. S3).

Performance measures were based on each child’s predicted probability when held out from model selection and estimation as an unseen ‘test case’. Discrimination (the model’s ability to accurately classify those with and without poor functioning) was visualized using the receiver operator characteristic (ROC) curve and quantified using the area under the curve (AUC; Steyerberg, 2009). AUC ranges from 0.5 (chance-level) to 1 (perfect) discrimination with the following proposed benchmarks (Hosmer, Lemeshow, & Sturdivant, 2013): <0.7 (poor); 0.7–0.8 (acceptable); 0.8–0.9 (excellent); 0.9–1.0 (outstanding).

Calibration was assessed by plotting predicted probabilities (grouped into equi-interval bins) against observed outcomes, where a 45° line indicates perfect agreement. A chi-square test of unreliability (*U*) determines whether each calibration plot’s intercept (calibration-in-the-large) and slope significantly differed from the ideal line (Fenlon, O’Grady, Doherty, & Dunnion, 2018).

Overall performance was assessed by the percentage of explained deviance (“pseudo r^2^”) and the Brier (1950) score or mean squared difference between predicted probabilities and actual binary outcomes. We scaled Brier scores by their maximum possible values, which vary based on the incidence of the outcome, ranging from 0% (non-informative) to 100% (perfect; Steyerberg, 2009).

Finally, we carried out two sensitivity analyses. First, we tested whether model performance and/or interpretability would improve if we allowed inclusion of a set of correlated data in our model. To do this, we re-estimated each model using elastic net regularization. Unlike LASSO, this regularized regression method selects or excludes sets of correlated variables, potentially allowing more predictors to be retained. Second, to test whether predictive ability was inflated by the presence of non-independent twin observations, we re-ran analyses in ten subsamples, each consisting of one twin per twin-pair.

## 3 Results

### 3.1 Descriptive statistics

Descriptive statistics of the predictor variables for severely victimized children are shown in Table 3. Compared to their non-victimised peers, individuals exposed to childhood victimization were at greater risk of psychosocial (OR = 2.01; 95% CI: 1.62, 2.51; *p* < 0.001) and economic (OR = 2.57; 95% CI: 2.03, 3.27; *p* < 0.001) disadvantage at age 18 years. However, despite these increased risks, 37% (*n* = 205) of victimized children did not develop psychosocial disadvantage and 52.6% (*n* = 289) did not develop economic disadvantage.

**Table 3:** Descriptive Statistics for Individuals Exposed to Childhood Victimization.

Predictor	Victimized Sample (*N* = 555-591)	
	*M*	*SD*
**Individual**		
IQ	91.69	14.80
Openness to experience	4.10	2.80
Conscientiousness	7.77	3.56
Extraversion	8.41	3.39
Agreeableness	8.73	1.86
Neuroticism	2.21	1.99
Attention-deficit/hyperactivity disorder symptoms	2.35	3.20
Conduct disorder symptoms	1.06	1.58
Anxiety symptoms	8.25	3.25
Depression symptoms	5.01	7.62
**Family**		
Maternal warmth	6.55	1.73
Sibling warmth	18.93	3.58
Adult involvement	23.32	3.97
Family history of psychopathology	0.50	0.28
**Community**		
Neighborhood crime victimization	1.22	1.53
Social cohesion	6.78	3.19
Status among peers	2.75	1.13

	*N*	%
**Individual**		
Female sex	294	49.8
Any self-harm/suicide attempt	38	6.6
1+ definite psychotic symptoms	67	11.6
**Family**		
Biological parents in household:		
Both parents always present	168	29.1
One parent absent at some point	352	61.0
Both parents always absent	57	9.9
Socioeconomic status:		
Low	304	51.4
Middle	164	27.8
High	123	20.8

*Note*. IQ = intelligence. For continuous variables, the mean (M) and standard deviation (SD) are given. For categorical variables, the number (N) and proportion (%) of victimized children within each category is presented.

### 3.2 Prediction modelling

Given the heterogeneity of outcomes within the victimized group, we developed two prediction models to predict whether or not a severely victimized child would have (i) psychosocial disadvantage and (ii) economic disadvantage at age 18 years. Regression coefficients (Table 4) were obtained by re-estimating each LASSO model following nested cross-validation using the mean optimal lambda from each of the 10 training loops.

**Table 4:** Unstandardized Regression Coefficients and Proportion of Deviance Explained for LASSO Models Predicting Psychosocial and Economic Disadvantage.

Predictor	Psychosocial disadvantage (*N* = 506)	Economic disadvantage (*N* = 503)
	*B*	*B*
Model intercept	1.83	7.03
**Individual**		
Female sex	0.05	−0.17
IQ	.	−0.05
Openness to experience	.	.
Conscientiousness	.	−0.04
Extraversion	.	−0.01
Agreeableness	.	−0.03
Neuroticism	.	−0.002
ADHD symptoms	.	0.10
CD symptoms	0.14	.
Anxiety symptoms	0.05	.
Depression symptoms	0.02	.
Self-harm/suicide attempt	0.07	.
Psychotic symptoms	.	.
**Family**		
Maternal warmth	−0.004	−0.10
Sibling warmth	−0.02	−0.03
Adult involvement	−0.06	.
Family history of psychopathology	0.31	.
Biological parents in household	.	.
SES	.	−0.28
**Community**		
Neighborhood crime victimization	0.08	.
Social cohesion	−0.02	.
Status among peers	−0.09	.

**Deviance explained (pseudo r^2^)**	8.8%	25.0%

*Note*. ADHD = Attention-Deficit/Hyperactivity Disorder. *B* = unstandardized regression coefficient; CD = Conduct Disorder; IQ = intelligence; LASSO = Least Absolute Shrinkage and Selection Operator; SES = socio-economic status. Predictor variables with no coefficient reported have been reduced to exactly zero and thus removed from the model. The formulae for calculating the predicted probability that a victimized individual will have psychosocial and economic disadvantage at age 18 are as follows: P (psychosocial disadvantage) = 1/(1+exp (− (1.83 + 0.05*female sex + 0.05*anxiety + 0.02*depression + 0.14*conduct disorder + 0.07*self-harm/suicide – 0.09*status among peers -0.004*maternal warmth – 0.02*sibling warmth – 0.06*adult involvement + 0.31*family history of psychopathology + 0.08*neighborhood crime victimization – 0.02*social cohesion))). P(economic disadvantage) **=** 1/(1+exp (− (7.03–0.05*IQ – 0.17*female sex – 0.04*conscientiousness – 0.01* Extraversion – 0.03*agreeableness – 0.002* neuroticism + 0.10*ADHD – 0.10*maternal warmth – 0.03*sibling warmth – 0.28*SES))).

#### 3.2.1 Psychosocial disadvantage

A total of 314 (62.1%) individuals had psychosocial disadvantage at age 18 years following childhood victimization. Based on a mean lambda tuning parameter of 0.019 (range = 0.014–0.025), 12 of the 22 predictors were retained in the model.

**Fig. 1:**
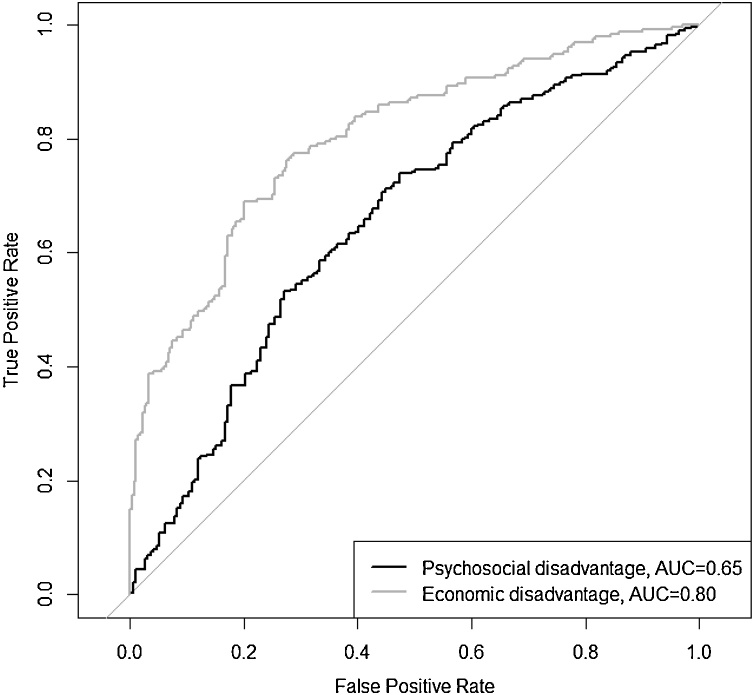
Receiver operating characteristic (ROC) curves for the model predicting psychosocial disadvantage and the model predicting economic disadvantage at age 18 years. True positive rate (sensitivity) = proportion of actual positive outcomes correctly identified as such. False positive rate (1 – specificity) = proportion of incorrectly-classified positive outcomes. Diagonal line represents chance-level (i.e. 50%) discrimination.

**Fig. 2:**
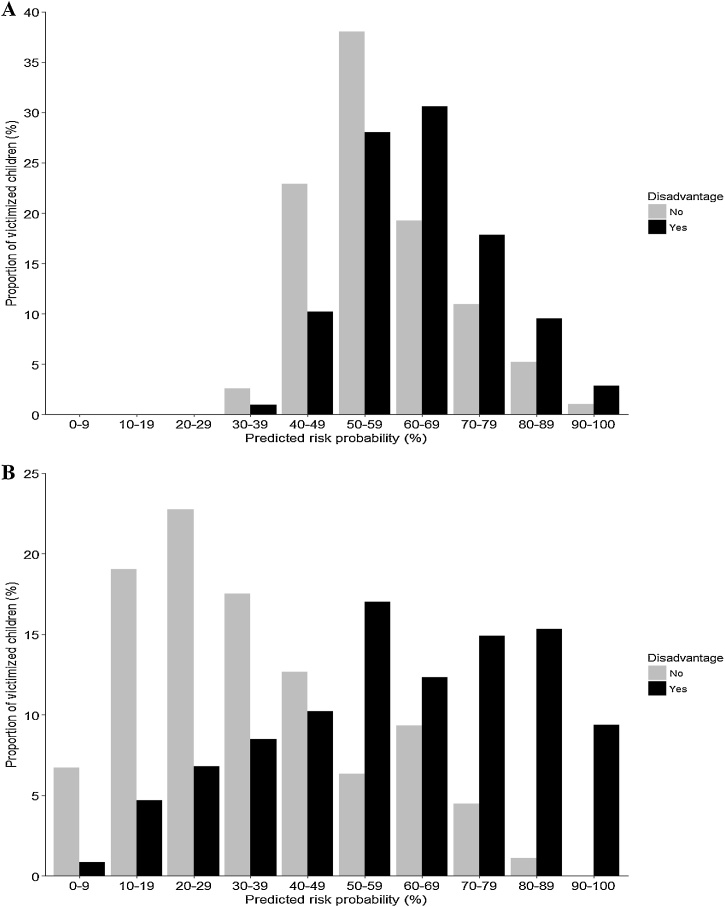
Frequency distributions of predicted risks among victimized children with and without psychosocial disadvantage (Panel A; *N* = 506) and economic disadvantage (Panel B; *N* = 503) at age 18 years.

Based on nested 10-fold cross-validation for internal validation, the model achieved an AUC of 0.65 (95% CI: 0.60, 0.70; Fig. 1). This indicates that the model performs better than random chance; there was a 65% probability that a randomly selected victimized individual with psychosocial disadvantage had a higher risk score than a randomly selected victimized individual without. The frequency distribution of predicted risk scores for individuals with and without psychosocial disadvantage are shown in Fig. 2, Panel A. Disadvantage occurs at a proportionally higher rate than no disadvantage from a predicted risk of 60% upwards. Model classification measures at a range of dichotomous risk score cut-offs are presented in Supplementary Table S4.

**Fig. 3:**
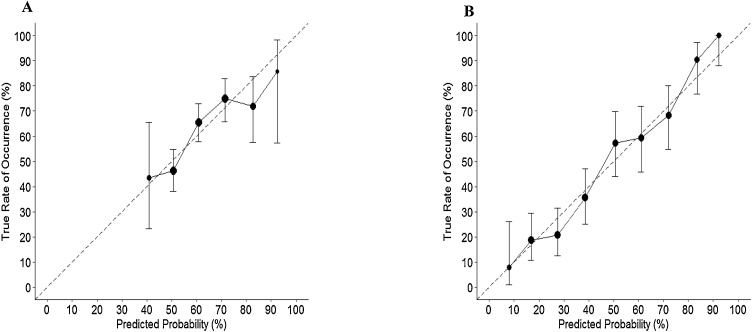
Calibration plots for the model predicting psychosocial disadvantage (Panel A) and economic disadvantage (Panel B) at age 18 years following childhood victimization. Plots show the degree of agreement between model-predicted risk probability and the true observed rate of occurrence. A well-calibrated model shows predictions lying on or around the 45˚ line which represents perfect calibration (shown here as a dotted line). Error bars represent 95% confidence intervals. The size of the data-point increases with the number of cases in that predicted probability bin.

This model was particularly well-calibrated within the range of 40–70% predicted risk probability (Fig. 3, Panel A) – within which 87% of the prediction fell (predicted risk probability median = 0.60; interquartile range = 0.53–0.70). Due to an absence of cases with a risk probability below 35% we were unable to assess calibration at low levels of risk. The calibration line (slope = 0.84, intercept = 0.07) was not significantly different from the perfect calibration line (*U* = −0.002, χ^2^(2) = 0.78, *p* = 0.677). In terms of overall model performance, the model explained 8.8% of the deviance and the scaled Brier score was 5.7%, suggesting that even though the model was fairly well-calibrated, there remained unexplained error around its predicted probabilities.

#### 3.2.2 Economic disadvantage

A total of 235 (46.7%) victimized children had economic disadvantage at age 18 years. Based on a mean lambda tuning parameter of 0.020 (range = 0.017–0.028), 10 predictors were retained.

Internal validation showed that the model achieved an AUC of 0.80 (Fig. 1; 95% CI: 0.76, 0.84), demonstrating that it discriminated well between victimized children who did and did not go on to have economic disadvantage at age 18 years. Fig. 2, Panel B shows the frequency distribution of predicted risk scores for individuals with and without economic disadvantage. Disadvantage occurs at a proportionally higher rate than no disadvantage from a predicted risk of 50% upwards. For model classification measures at a range of thresholds see Supplementary Table S5.

The calibration plot (Fig. 3, Panel B) suggests a high degree of consistency between model-predicted and observed risks for developing economic disadvantage across the range of risk scores. Indeed, the slope and intercept of the calibration line (slope = 1.11, intercept = 0.01) was not significantly different from the perfect calibration line (*U* = −0.002, χ^2^(2) = 1.12, *p* = 0.570). In terms of overall model performance, the model explained 25% of the deviance and the scaled Brier score was 27.8%, indicating a notable reduction in mean squared error compared to an un-informative model.

### 3.3 Sensitivity analyses

#### 3.3.1 Regularization penalty

For both psychosocial and economic disadvantage, model coefficients and performance statistics (Supplementary Tables S6 and S7) from nested 10-fold cross-validation using a less restrictive form of regularization (i.e., ‘elastic net’) were similar to those of the LASSO models. This suggests that predictive ability was not worsened by our use of the most restrictive form of regularization.

#### 3.3.2 Non-independence of twins

Average cross-validated performance statistics for each outcome across 10 random single-twin subsamples (*n* = 302–305) resembled those obtained using the full victimized sample (Supplementary Tables S8 and S9) suggesting that our use of twin (non-independent) data did not significantly bias predictive accuracy.

## 4 Discussion

This study demonstrates that factors known to be associated with poor functioning following childhood victimization can be statistically modelled to predict individual risk of psychosocial and economic disadvantage at the transition to adulthood. Our results show that a different combination of factors is needed to predict victimized children’s risk for psychosocial disadvantage than economic disadvantage at age 18 years. Psychosocial disadvantage was best predicted by a combination of individual (sex, conduct disorder, anxiety, self-harm/suicide), family (maternal warmth, sibling warmth, adult involvement, family history of psychopathology) and community (neighborhood crime victimization, social cohesion, status among peers) factors, whereas economic disadvantage was predicted by individual (IQ, sex, conscientiousness, extraversion, neuroticism, ADHD) and family (maternal warmth, sibling warmth, SES) factors but no community factors. Both models were well-calibrated and performed within the range of established, externally-validated, calculators for cardiovascular disease and cancer (e.g. DeFilippis et al., 2015; Wang et al., 2018), although only the economic disadvantage model showed good overall performance. Whilst our model predicting psychosocial disadvantage was able to accurately predict a victimized child’s probability of having the poor outcome, it was less able to determine whether they would actually have psychosocial disadvantage at age 18 years or not. Ideally risk prediction models should be both well-calibrated and have high discrimination, however, the accuracy of the predicted probabilities (i.e. calibration) is, arguably, more important for models with a prognostic, rather than diagnostic, purpose such as ours (Cook, 2007). Even though not all of the victimized individuals identified by our models as being at high risk for poor functioning will go on and have that outcome, in this setting providing support and intervention to all of those who are at high risk may be a beneficial strategy.

Thus, our results are a promising step towards supporting practitioners to predict which victimized children will go on to have the poorest functional outcomes. Ultimately, practitioners may, for example, use the risk calculator as a screening tool when they assess a victimized child and use the resulting individualized score to help guide their decision as to whether that child may benefit from more intensive intervention. In this way the risk calculator could help practitioners to identify who to target with preventative interventions (i.e. those who are at highest risk). It does not, however, inform what the intervention itself should be; this would be illuminated by a separate line of research exploring the factors that predict which victimized children identified as being at high risk for poor functioning do and do not go on to have such outcomes, an important avenue for future research.

Our work reflects a growing interest in predictive analytics, complementing attempts within clinical and child protection domains to use algorithms to identify patients at risk of domestic abuse (Reis, Kohane, & Mandl, 2009) and children at risk of maltreatment (Cuccaro-Alamin, Foust, Vaithianathan, & Putnam-Hornstein, 2017; Hurley, 2018; McIntyre & Pegg, 2018). Moreover, similar methods have been used to predict individual risk of developing psychopathology at age 18 years following exposure to childhood victimization (Meehan et al., 2019) which, together with the current study, demonstrates the applicability of prediction modelling to several different outcomes within groups of victimized children.

Some limitations of our study warrant acknowledgement. First, because of the relatively small sample, we refrained from studying more complex models. For example, we included sex as a predictor but did not explore its interaction with other predictors and we did not explore prediction of multiple functional difficulties. Consistent with recommendations (e.g., Steyerberg & Vergouwe, 2014), however, we selected predictors based on existing literature and this did not provide a strong rationale for the inclusion of particular interaction terms. Second, sample size precluded separate investigation of specific types of victimization. It is possible that the resulting risk calculator, and its predictive performance, might vary according to the type of victimization exposure. However, children exposed to one type of severe victimization often also experience one or more other types (Fisher et al., 2015). Third, we utilized a twin sample and the extent to which findings from twins can be generalized to non-twins is sometimes contested. However, prevalence rates for childhood victimization in the E-Risk cohort are comparable to recent UK population estimates (Radford et al., 2013), the sample is representative of UK families in terms of geographic and socioeconomic distribution, and our sensitivity analyses indicated that using twins had minimal impact on our internal validation results. Fourth, we developed and internally validated our risk calculator using a UK population-representative sample, therefore, its cross-cultural validity remains unknown, and it also may not generalize to cases of extreme victimization (e.g. children in foster care). Relatedly, in the E-Risk sample there was sparse representation for victimized children at low risk (<35%) of psychosocial disadvantage. Whilst this may indeed reflect the general population, we caution that we were unable to adequately test the calibration of our model at this level. Fifth, we focus on predicting risk for poor functioning at age 18 years and, therefore, our risk calculator is not informative about functioning prior, or subsequent, to this. Individuals exposed to childhood victimization may show variability in functioning over time (DuMont, Widom, & Czaja, 2007) as new experiences and life events unfold that confer vulnerability or resilience. Moreover, at 18 years old many remain financially dependent on their parents (e.g. living at home) which may reduce the immediate impact of their economic disadvantage. Nonetheless, at the cusp of adulthood, this outcome is likely an indicator of that young person’s reduced economic opportunities in the future. Sixth, although discrimination was excellent for the model predicting economic outcomes, it was poor for the model predicting psychosocial disadvantage. We posit that this may be due, in part, to the more subjective nature of the psychosocial outcome measures relative to the economic measures. Finally, we did not have a sample available for external validation. Unlike previous studies we assessed the predictive performance of unseen cases using nested cross-validation as recommended by Hastie et al. (2009). However, internal validation likely overestimates our models’ performance in other samples; therefore, assessing model predictive performance using independent data is essential. It will also be important to ensure in the future that the model is ecologically valid – that predictors are available to practitioners in their work settings, and thus, can be applied in practice.

Despite these limitations, our work highlights the potential of prediction modelling to support practitioners (e.g., social workers, educators, clinicians) working with victimized children. Currently, such practitioners make decisions based on their clinical experience in judging children’s risk for developing poor outcomes. Our findings, however, demonstrate the potential utility of risk calculators to assist their identification of the most vulnerable children and, thus, support them to effectively target interventions to promote positive functional outcomes at the crucial transition to adulthood. This is especially important in a context of scarce resources.

## Declaration of Competing Interest

None.
