# Supplementary material for: Development of an individualized risk calculator for poor functioning in young people victimized during childhood: A longitudinal cohort study
Source: Child Abuse Negl. 2019 Dec;98:104188. doi: 10.1016/j.chiabu.2019.104188 (PMC6905153; doi:10.1016/j.chiabu.2019.104188)
Supplement: Supplementary file 1 [file mmc1.docx]

**Supplementary Materials**

**The Environmental Risk (E-Risk) Cohort**

The E-Risk Study tracks 2,232 twins in 1,116 same-sex pairs born in England and Wales in 1994-1995. At inception, the sample was constructed from the Office for National Statistics (ONS) multiple-birth register to accurately represent the full range of Britain’s socioeconomic conditions. We under-recruited twins born to older, well-educated mothers who used assisted reproduction, to avoid the middle-class bias typical of twin studies. We over-recruited twins born to teenaged mothers, to replace poor families because poor families were disproportionately lost to the ONS register due to nonresponse. Figure S1 shows E-Risk families’ addresses are a near-perfect match to the deciles of the UK’s 2015 Lower-layer Super Output Area (LSOA) Index of Multiple Deprivation (IMD) which averages 1,500 residents; approximately 10% of the cohort fills each of IMD’s 10% bands.

**Childhood Victimization**

Lifetime exposure to several types of victimization was assessed repeatedly when the children were 5, 7, 10, and 12 years of age and dossiers have been compiled for each child with cumulative information about exposure between birth and age 12 years to domestic violence between the mother and her partner; frequent bullying by peers; physical abuse by an adult; sexual abuse; physical neglect; and emotional abuse/neglect. The E-Risk team has previously reported evidence on the reliability and validity of the measures of domestic violence (Moffitt et al., 1997), bullying (Arseneault et al., 2006; Shakoor et al., 2011), physical and sexual abuse (Jaffee, Caspi, Moffitt, & Taylor, 2004; Jaffee, Caspi, Moffitt, Polo-Tomas, & Taylor, 2007), emotional abuse/neglect (Danese et al., 2017), and physical neglect (Fisher et al., 2015). All the component measures are outlined briefly below.

**Physical domestic violence.** Mothers reported about perpetration of and victimization involving 12 forms of physical violence (e.g., slapping, hitting, kicking, and strangling) from the Conflict Tactics Scale (Straus, 1990), on three assessment occasions during the child’s first decade of life (when the children were 5, 7, and 10 years of age). Reports of either perpetration or victimization constituted evidence of physical domestic violence. Families in which no physical violence took place were coded as 0 (55.2%); families in which physical violence took place on one occasion were coded as 1 (28.0%); and families in which physical violence took place on multiple occasions were coded as 2 (16.8%).

**Bullying by peers.** Experiences of victimization by bullies were assessed using both mothers’ and children’s reports. During the interview, the following standard definition of bullying was read out: “Someone is being bullied when another child (a) says mean and hurtful things, makes fun, or calls a person mean and hurtful names; (b) completely ignores or excludes someone from their group of friends or leaves them out on purpose; (c) hits, kicks, or shoves a person, or locks them in a room; (d) tells lies or spreads rumors about them; and (e) other hurtful things like these. We call it bullying when these things happen often, and when it is difficult to make it stop. We do not call it bullying when it is done in a friendly or playful way.”

Mothers were interviewed when children were 7, 10, and 12 years old and asked whether either twin had been bullied by another child, responding ‘never’, ‘yes’, or ‘frequently’. We combined mothers’ reports at child age 7 and 10 to derive a measure of victimization during primary school. Mothers’ reports when the children were 12 years old indexed victimization during secondary school. During private interviews with the children when they were 12 years old, the children indicated whether they had been bullied by another child during primary or secondary school. When a mother or a child reported victimization, the interviewer asked her to describe what happened. Notes taken by the interviewers were later checked by an independent rater to verify that the events reported could be classified as instances of bullying operationally defined as evidence of (a) repeated harmful actions, (b) between children, and (c) where there is a power differential between the bully and the victim (Shakoor et al., 2011). Although interrater reliability between mothers and children was only modest (*k* = 0.20–0.29), reports of victimization from both informants were similarly associated with children’s emotional and behavioral problems, suggesting that each informant provides a unique but meaningful perspective on bullying involvement (Shakoor et al., 2011). We thus combined mother and child reports of victimization to capture all instances of bullying victimization for primary and secondary school separately: reported as not victimized by both mother and child; reported by either mother or child as being occasionally victimized; and reported as being occasionally victimized by both informants or as frequently victimized by either mother or child or both (Bowes et al., 2013). We then combined these primary and secondary school ratings to create a bullying victimization variable for the entire childhood period (5–12 years). Children who were never bullied in primary or secondary school or occasionally bullied during one of these time periods were coded as 0 (55.5%); children who were occasionally bullied during primary and secondary school, or frequently bullied during one of these time periods were coded as 1 (35.6%); and children who were frequently bullied at both primary and secondary school were coded as 2 (8.9%).

**Physical and sexual harm by an adult.** We assessed childhood physical and sexual harm in the E-Risk Study using an approach that resembles the process undertaken by child protection agencies. Essentially this is a two-stage process. In child protection, professionals such as teachers working with children typically raise concerns if they observe signs or symptoms or if they become aware of risk that children are victims of violence. When concerns are raised, child protection officers then review the concerns and evaluate them in the context of information previously gathered on that child or family in order to determine the likelihood that abuse has taken place. In the E-Risk Study, research workers visited the home in pairs, and were extensively trained to detect signs of abuse or neglect. Each time the two research workers visited a home, they interviewed the mother using a structured interview about child harm, tested the children, and observed the family environment using the Home Observation for Measurement of the Environment (HOME) (Bradley & Caldwell, 1977). If either research worker had any concerns, they flagged up the case for review. Immediately after each home visit, a review was performed if a family was flagged. In addition, at each wave, any family who had been flagged on a prior wave of the study was automatically reviewed again. The reviews were performed independently by at least 2 clinical psychologists or psychiatrists and were based on comprehensive dossiers compiled across multiple home visits for each study member during the course of the ongoing longitudinal study.

An unusual feature of the E-Risk study’s assessment is that we repeatedly interviewed mothers on four occasions over the years, which allowed them to build confidence in the research team. Also, we were able to reassure mothers that if harm to the child was ongoing and had to be reported by us, reporting would be managed through a trusted familiar professional, namely the family’s registered GP. As the children grew older, some mothers who were initially reluctant to reveal abuse to us, divulged details of severe abuse at a later interview.

At age 5, assessments were based on the standardized clinical protocol from the MultiSite Child Development Project (Dodge, Bates, & Pettitt, 1990; Lansford et al., 2002). At ages 7, 10, and 12 this interview was modified to expand its coverage of contexts for child harm. Interviews were designed to enhance mothers’ comfort with reporting valid child maltreatment information, while also meeting researchers’ responsibilities for referral under the UK Children Act. Specifically, mothers were asked whether either of their twins had been intentionally harmed (physically or sexually) by an adult or had contact with welfare agencies. If caregivers endorsed a question, research workers made extensive notes on what had happened, and indicated whether physical and/or psychological harm had occurred. Under the UK Children Act, our responsibility was to secure intervention if maltreatment was current and ongoing. Such intervention on behalf of E-Risk families was carried out with parental cooperation in all but one case. No families left the study following intervention.

Over the years of data collection, the study developed a cumulative profile for each child, comprising the caregiver reports, recorded debriefings with research workers who had coded any indication of maltreatment at any of the successive home visits, recorded narratives of the successive caregiver interviews, and information from clinicians whenever the Study team made a child-protection referral. Each time we visited a home, the research workers flagged concerns, and if there was sufficient evidence to code definite harm then we did so. If evidence only met the level of probable harm, we kept an “ongoing concern list” and if, at a later wave, there was continued evidence of probable harm, or new evidence, the code was upgraded to definite harm. The profiles were reviewed at the end of the age-12 phase by at least two clinical psychologists or psychiatrists. Initial inter-rater agreement between the coders was 90% in cases for whom maltreatment was identified (100% for cases of sexual abuse), and discrepantly coded cases were resolved by consensus review. These were coded as: 0 = no physical harm at any age; 1 = probable physical harm at any age; and 2 = definite physical harm at any age. There were 15.0% of the children coded as probably being exposed to physical harm and 5.1% as definitely physically harmed by 12 years of age. There were 0.8% of the children coded as probably exposed to sexual abuse, and 0.7% as definitely exposed to sexual abuse by 12 years of age.

**Emotional abuse and neglect.** These forms of maltreatment were coded from research workers’ narratives of the home visits at ages 5, 7, 10, and 12. We coded quite severe examples of parental behavior observed. For example, a mother who had schizophrenia screamed and swore at the children throughout the home visit. As another example, a father who was drunk during the home visit repeatedly spoke abusively to the children in front of the research workers. We found that coders could not empirically separate emotional abuse and emotional neglect in a reliable way and thus such experiences were coded together as emotional abuse/neglect. Inter-rater agreement between the coders exceeded 85% for cases with emotional abuse and neglect, and discrepant cases were resolved by consensus review. Children with no evidence of emotional abuse/neglect were coded as 0 (88.5%), those where there was some indication of emotionally inappropriate/potentially abusive or neglectful behavior were coded as 1 (8.5%), and where there was evidence of severe emotional abuse/neglect the children were coded as 2 (3.0%).

**Physical neglect.** The cumulative observations of the physical state of the home environment documented by the interviewers during home visits to the twins at ages 5, 7, 10, and 12 were reviewed by two raters for evidence of physical neglect. This was defined as any sign that the caretaker was not providing a safe, sanitary, or healthy environment for the child. This included the child not having proper clothing or food, as well as grossly unsanitary home environments. (However, this did not include a family living in a crime-ridden neighborhood for economic reasons.) Initial interrater agreement between the coders exceeded 85%, and discrepantly coded cases were resolved by consensus review. Children with no evidence of physical neglect were coded as 0 (90.9%), those for whom there was an indication of minor physical neglect were coded as 1 (7.1%), and where there was evidence of severe physical neglect the children were coded as 2 (2.0%).

**Age-18 Functional Outcomes**

**Low educational achievement.** Participants were asked whether they had any of the following educational qualifications: 1+ GCSEs (any grades); 5+ GCSEs (grades A, B or C); A level, AS or S level, or Access Course; 2+ A levels, or 4+ AS or S levels, or any HSCs; NVQ or other vocational qualification; City and Guilds, RSA, OCR, BTEC or Edexcel; or other qualification. Based on the Qualifications and Credit Framework (QCF) responses were coded as ‘0’ no qualification; ‘1’ level 1 qualification (GCSE at grades D-G); ‘2’ level 2 qualification (GCSE at grades A*-C) or ‘3’ level 3 qualification (A-Level). In the present study low educational achievement was dichotomized to represent level 1 qualification or less (1) versus level 2/3 qualification (0). A total of 21.9% of E-Risk Study participants had low educational achievement.

**Not in Education, Employment or Training (NEET).** Participants were classified as NEET if they reported in the age-18 interview that they were neither studying, nor working in paid employment, nor pursuing a vocational qualification or apprenticeship training (Goldman-Mellor et al., 2016). Participants were queried to ensure that NEET status was not simply a function of being on summer holiday, or of being a parent. This operationalization of NEET status follows that used by the UK Office of National Statistics and the International Labour Organization (Office for National Statistics, 2013). In the E-Risk cohort, 11.6% of participants were NEET, matching UK national NEET figures (Office for National Statistics, 2014).

**Parenthood.** Information on parenthood was obtained in the age-18 interview by asking participants about the outcome of any previous pregnancies that they had experienced (for girls) or caused (for boys). Girls were additionally asked whether they were currently pregnant. Participants were classified as parents if a previous pregnancy had resulted in a live birth or if they were currently pregnant. 2.9% of girls and 1.1% of boys had experienced or caused at least one pregnancy that had resulted in a live birth and 0.9% of girls were pregnant at the age-18 interview. The observed rates match the UK national figures on live births for this age group (Office for National Statistics, 2016).

**Cautions and convictions.** Official records of participants’ cautions and convictions were obtained through UK Police National Computer (PNC) record searches conducted in cooperation with the UK Ministry of Justice. E-Risk participants gave their written informed consent at age 18 years for the search of police records to be undertaken. A total of 2060 twins gave informed consent for the search at age-18 interview (99.3% of those taking part at age-18; 92.3% of the original cohort). This search was only undertaken when this consent was provided. The PNC matching algorithm, which is the same as used in law enforcement, used first, middle, and last name, date of birth, and home address. Ambiguous matches were checked by hand. Records include complete histories of cautions and convictions for participants cautioned or convicted in the UK beginning at age 10 years, the age of criminal responsibility. A criminal offence in the PNC is an offence under the Criminal Law code of the UK. Reprimands, warnings, driving offences and public order offences are not considered convictions for crimes. Cautions and convictions were recoded into a binary variable to reflect whether participants had been cautioned or convicted (1) or not (0). A total of 10.8% of E-Risk participants had been cautioned or convicted.

**Adolescent poly-victimization.** Participants reported their experiences of victimization between 12 and 18 years using the Juvenile Victimization Questionnaire (JVQ) (Finkelhor et al., 2011; Hamby et al., 2004), adapted as a clinical interview. Full details of this measure have been reported previously (Fisher et al., 2015). In brief, our adapted JVQ comprised 45 questions covering different forms of victimization grouped into seven categories: crime victimization, peer/sibling victimization, Internet/mobile phone victimization, sexual victimization, family violence, maltreatment, and neglect. Each JVQ question was asked for the period “since you were 12”. Participants were given the option to say “yes” or “no” as to whether each type of victimization had occurred in the reporting period. If an experience was endorsed, follow-up questions were asked concerning how old the participant was when it (first) happened, whether the participant was physically injured in the event, whether the participant was upset or distressed by the event, and how long it went on for (by marking the number of years on a Life History Calendar; Caspi et al., 1996). In addition, the interviewer wrote detailed notes based on the participant’s description of the worst event. All information from the JVQ interview was compiled into victimization dossiers. Using these dossiers, each of the seven victimization categories was rated by an expert in victimology and 3 other members of the E-Risk team who were trained on using the rating criteria. Ratings were made using a 6-point scale: 0 = not exposed, then 1–5 for increasing levels of severity. Ratings of 4 or 5 were indicative of exposure to severe victimization. The number of different types of severe victimization experienced by each participant were then summed to create a score of adolescent poly-victimization (i.e. experiencing multiple types of severe victimization). We dichotomized this to reflect no poly-victimization (coded 0 = 0 or 1 type of severe victimization) versus poly-victimization (coded 1 = 2 or more types of severe victimization). A total of 16.2% of children reported adolescent poly-victimization (*n* = 334).

**Social isolation.** Social isolation was measured using the Multidimensional Scale of Perceived Social Support (MSPSS), which assesses individuals’ access to supportive relationships with family and friends (Zimet, Dahlem, Zimet & Farley, 1988) with 12 items consisting of statements such as “There is a special person who is around when I am in need” and “I can count on my friends when things go wrong.” Participants rated these statements as “not true” (0), “somewhat true” (1) or “very true” (2). We reversed the scoring of the items so that higher scores reflected greater isolation. We defined social isolation as being among the 20% highest scoring participants.

**Low life satisfaction.** Participants’ life satisfaction was assessed using the Satisfaction with Life Scale (Diener, Emmons, Larsen & Griffin, 1985) with 5 items including “The conditions of my life are excellent” and “I am satisfied with my life”. The response format was a 5-point scale ranging from “strongly disagree” (1) to “strongly agree” (5). We reversed the scoring of the items so that higher scores reflected lower life satisfaction. We defined low life satisfaction as being among the 20% highest scoring participants.

**Loneliness.** Participants’ feelings of loneliness were assessed using four items from the UCLA loneliness scale (Version 3; Russell, 1996)*:* “How often do you feel that you lack companionship?”, “How often do you feel left out?”, “How often do you feel isolated from others?” and “How often do you feel alone?” The full UCLA Scale consists of 20 items; however, a previous study has shown that a short form of the scale has adequate validity for inclusion in large-scale studies (Hughes, Waite, Hawkley, & Cacioppo, 2004). The items were rated “hardly ever” (0), “some of the time” (1) or “often” (2). The items were administered as part of a self-completed computer-based questionnaire. We summed the items to produce a total loneliness score and defined loneliness as being among the 20% highest scoring participants.

**Low sleep quality.** Sleep quality was measured using the Sleep Quality Index (PSQI; Buysse, Reynolds, Monk, Berman, & Kupfer, 1989). The PSQI consists of 18 self-report items relating to individuals’ sleep patterns and different forms of sleep impairment in the past month. Questions tap a range of aspects of sleep quality and can be used to derive seven component scores (subjective sleep quality, sleep latency, sleep duration, habitual sleep efficiency, sleep disturbances, use of sleep medication and daytime dysfunction) each scored from 0 to 3. These were summed to produce a global score ranging from 0 to 21 with higher scores reflecting lower sleep quality. We defined low sleep quality as being among the 20% highest scoring participants.

**Childhood Predictors**

Protective factors associated with a better life following childhood maltreatment identified by a recent systematic review of the literature (Meng, Fleury, Xiang, Li & D’Arcy, 2018) were mapped to variables measured in the E-Risk Study between child ages 5 and 12 years (Table S3). From those available we selected a maximum of 22 (to ensure a minimum of 10 events per predictor) taking into consideration the practicability of measures for clinicians/practitioners and ensuring representation from individual, family and community factors. In addition to those factors identified by the systematic review, we included sex and childhood measures of psychopathology (anxiety, depression, conduct disorder, attention deficit hyperactivity disorder (ADHD), self-harm/suicide, psychotic symptoms) as we expected these to be related to many of the factors identified and studies have shown them to impact functional outcomes. For example, conduct disorder has been associated with a higher risk of later criminal offending, low life satisfaction, social isolation and early parenthood (Wertz et al., 2018); and early-onset mental health difficulties related to lower educational attainment (Fletcher, 2010; Woodward & Fergusson, 2001) and NEET status (Goldman-Mellor et al., 2016).

**Individual-level predictors.**

***Sex*** was reported by mothers at study baseline (1 = male; 2 = female).

***IQ*** was tested at age 12 using a short version of the Wechsler Intelligence Scale for Children – Revised (WISC-R, Wechsler, 1974) which comprised two subtests (Matrix Reasoning and Information). We prorated study members’ IQ according to the method recommended by Sattler (2008). IQ scores ranged from 48 to 143 (*M* = 96.25, *SD* = 14.98) in the E-Risk sample.

***Personality*** was measured using the child version of the Big Five Inventory (John & Srivastava, 1999). Study interviewers rated each twin at the end of their two- to three-hour home visit when children were aged 12. They rated each twin on the presence of 44 personality characteristics along a three-point rating scale (0 = ‘no’; 1 = ‘a little/somewhat’; 2 = ‘yes’). From these responses, separate scores were derived for each of the ‘Big Five’ personality dimensions: openness to experience (5 items e.g. perceptive and curious; *M* = 4.31, *SD* = 2.76), conscientiousness (6 items e.g. focused and diligent; *M* = 8.52, *SD* = 3.23), extroversion (6 items e.g. gregarious and talkative; *M* = 8.28, *SD* = 3.54), agreeableness (5 items e.g. considerate and trusting; *M* = 8.94, *SD* = 1.70), and neuroticism (5 items e.g. fearful and touchy; *M* = 2.08, *SD* = 1.84).

***Attention-deficit/hyperactivity disorder (ADHD) symptoms*** were measured at age 12 using mother and teacher reports on 18 symptoms of inattention and hyperactivity-impulsivity, drawn from the Achenbach (1991a, 1991b) family of instruments. We averaged the mother- and teacher-rated symptom scores (*M* = 1.47, *SD* = 2.57).

***Conduct disorder (CD) symptoms*** at age 12 were derived from mother and teacher reports of child behavioral problems using the Achenbach (1991a, 1991b) family of instruments and additional DSM-IV items. 14 of 15 DSM-IV criteria were assessed, covering aggressive and non-aggressive conduct problems, deceitfulness or theft, and rule violations (‘forced sexual activity’ was excluded as it was considered age-inappropriate). Mother- and teacher-rated symptom counts were averaged (*M* = 0.61, *SD* = 1.10).

***Anxiety symptoms*** were assessed when children were aged 12 via private interviews using the 10-item version of the Multidimensional Anxiety Scale for Children (MASC; March, Parker, Sullivan, Stallings & Conners, 1997). Items were summed to indicate severity of anxiety (*M* = 7.62, *SD* = 3.04).

***Depression symptoms*** were assessed when children were aged 12 via private interviews using the 27-item Children’s Depression Inventory (CDI; Kovacs, 1992). Items were summed to indicate severity of depression (*M* = 3.11, *SD* = 5.32).

***Self-harm and suicide attempts*** were captured by asking mothers whether each twin had ever deliberately harmed him/herself or attempted suicide in the previous six months, as part of a face-to-face interview when the children were aged 12. Mothers who responded positively to this question were asked to provide a description of the event(s). An independent rater blind to other data subsequently used the interview notes to verify that the description provided was clearly an act of self-harm (Fisher et al., 2012). We asked only mothers, and not children, to report the child’s self-harm because of ethical considerations. From this, 2.9% (*n* = 62) of Study members had displayed any self-harming or suicidal behavior. Examples of self-harming behaviors included cutting and biting arms, pulling out clumps of hair, banging head against walls, and attempted suicides by strangulation.

***Psychotic symptoms*** were evaluated in private interviews by mental health trainees or professionals when children were aged 12. This interview has been described in detail previously (Polanczyk et al., 2010). To summaries, each child was privately interviewed about seven psychotic symptoms pertaining to delusions and hallucinations, with items including “have other people ever read your thoughts?”, “have you ever thought you were being followed or spied on?” and “have you ever heard voices that other people cannot hear?” A conservative approach was taken in designating a child's report as a symptom. First, the interviewer probed responses using standard prompts designed to discriminate between experiences that were plausible (e.g., “I was followed by a man after school”) and potential symptoms (e.g., “I was followed by an angel who guards my spirit”) and wrote down the child's narrative description of the experience. Second, validity of symptoms was verified by a psychiatrist expert in schizophrenia, a psychologist expert in interviewing children, and a child and adolescent psychiatrist. Third, because children were twins, experiences limited to the twin relationship (e.g., “My twin and I often know what each other are thinking”) were coded as ‘not a symptom’. Children were only classified as experiencing psychotic symptoms if they reported at least one definite symptom. At age 12, 5.9% (*n* = 125) of children reported experiencing at least one definite psychotic symptom.

**Family-level predictors.**

***Maternal warmth*** was assessed using procedures adapted from the Five-Minute Speech Sample method (Magaña et al., 1986). Mothers were asked to speak for 5 min about each of their children when they were aged 5 and again at age 10. The speech samples were audiotaped and coded by two independent raters, blind to all other E-Risk data who were shown to have good inter-rater reliability (*r* =.90). The warmth expressed by the mother in their interview about the child was assessed by the tone of voice, spontaneity, sympathy and/or empathy towards he child. Warmth was coded on a six-point scale from no warmth (0; complete absence of warmth) to high warmth (5; definite warmth, enthusiasm, interest in, and enjoyment of the child). As scores for maternal warmth at ages 5 and 10 were significantly correlated (*r* = 0.37, *p* < .001) these were summed to create a single ‘age 5-10’ score (*M* = 6.96, *SD* = 1.64).

***Sibling warmth*** was measured by asking mothers a series of questions about the quality of their children’s relationship with one another when the children were aged 7 and 10 (Jaffee, Caspi, Moffitt, Polo-Tomás, & Taylor, 2007). Mothers responded on a three-point scale to six questions (e.g., “do your twins love each other,” “do both your twins do nice things for each other”). Internal consistency at age 7 was α = 0.77 and at age 10 was α = 0.80. As age-7 and age-10 scores were highly correlated (*r* = 0.57, *p* < .001) these were summed to create a single ‘age 7-10’ composite score (*M* = 19.92, *SD* = 3.35).

***Adult involvement.*** The presence of a supportive adult was assessed at age 12 when children were asked questions about whether they had a stable adult figure to rely on for basic needs and support (e.g., “there is an adult who I can tell almost anything to,” “there is an adult who I can go to if I am in trouble”). These were coded on a three-point scale (0 = ‘not true’ to 2 = ‘definitely true). We derived a total score by summing responses to 13 items (internal consistency: α = 0.85; *M* = 23.78, *SD* = 3.44). It should be noted that these questions did not ask the child to specify who the adult was, and thus, this could have been someone within or outside of their family.

***Family history of psychopathology*** was assessed when children were aged 12. In private interviews, the twins’ mothers reported on her own history of DSM disorders, along with that of her biological mother, father, sisters, and brothers, as well as the twins’ biological father (Milne et al., 2009). This was converted to a proportion (0–1) of family members with a history of psychiatric disorder (*M* = 0.37, *SD* = 0.27).

***Number of biological parents in household.*** The twins’ living arrangements up to age 10 were assessed by asking mothers whether and when the twins’ biological father was living with the twins using a life history calendar (LHC), a visual method that facilitates the accurate recall of life events and their timing and duration (Caspi et al., 1996). Children were categorized as follows: ‘constantly lived with both their biological mother and father’ (52%; *n* = 1,108); ‘biological father absent at some point’ (40.7%; *n* = 868); ‘biological mother absent at some point’ (1.1%; *n* = 24); and ‘never living with both their biological parents (6.2%; *n* = 132). Due to the low prevalence of ‘biological mother absent’, this response was combined with ‘biological father absent’ and recoded to reflect the number of biological parents in the household: 0 (never lived with both biological parents; *n* = 132), 1 (one biological parent absent at some point; *n* = 892), 2 (both biological parents consistently present; *n* = 1,108).

***Socio-economic status (SES)*** was defined at age 5 using a standardized composite of parental income (i.e., total household income), education (i.e., highest parent qualification), and occupation (i.e., highest parent occupation). These three SES indicators were highly correlated (*r* = 0.57-0.67) and loaded significantly onto one latent factor (Trzesniewski et al., 2006). The population-wide distribution of this latent factor was then divided into tertiles (i.e., low-, medium-, and high-SES).

**Community-level predictors.**

***Neighborhood crime victimization*** was assessed when children were aged 5 by asking mothers whether they or their family had been the victim of a violent crime (e.g. mugging, assault), a burglary, or a theft in the neighborhood. The three items (each coded 0–2) were summed for each mother with higher scores indicating greater crime victimization (*M* = 0.92, *SD* = 1.31).

***Social cohesion*** was assessed using 5 items when the children were aged 5 by asking

mothers whether their neighborhood was close-knit, whether neighbors shared values, whether neighbors trusted and got along with each other, and whether neighbors were willing to help each other (Sampson, Raudenbush, & Earls, 1997). We derived a total score by summing these 5 items (internal consistency: α = 0.83) such that higher scores indicated greater social cohesion (*M* = 7.61, *SD* = 2.74).

***Status among peers.*** As part of a self-completed computer-based questionnaire at age 12, children reported on their perceived status among similar-aged peers. They were presented with an image of a target, comprising five concentric circles and asked to imagine that this represented other children about their age (both within and outside school). In the task instructions, the central circle was described as representing people who are ‘the center of attention and who are the most important – they usually have a lot of friends’. In contrast, the outermost circle represented people who ‘get the least attention, are not so important – they probably don’t have enough friends’. Children were asked to indicate which of the circles they felt they were in. Initial responses were reversed-scored such that they ranged from 0 (outermost circle; lowest peer status) to 4 (center circle; highest peer status; *M* = 2.87, *SD* = 0.97). A pilot study was conducted to validate this task among 49 children aged 10 and 11 years and found a correlation of 0.42 (*p* = .003) with the total score for the Children’s Loneliness Questionnaire (Asher & Wheeler, 1985).

**Statistical Analyses**

**Model development.** Using the *glmnet* package in R (Friedman, Hastie, & Tibshirani, 2010), separate prediction models were estimated for psychosocial and economic disadvantage via regularized logistic regression. Common explanatory approaches (e.g. logistic regression models), which provide an estimate of average (rather than individual) risk often display ‘over-fitting’; that is, they are overly specific to the unique structure and idiosyncrasies of the data on which they were developed, including any noise or error variance (see Figure S2). As a result, estimates of model performance are often over-optimistic, and frequently deteriorate once the model is applied to new data. To combat this, regularized regression places an additional constraint or penalty on the error term of the logistic regression equation (i.e., the binomial deviance), reducing the variance in the predicted values of model coefficients in an effort to mitigate over-fitting and promote generalizability to new or future observations. Importantly, the degree of penalty is determined by maximizing the predictive ability in the unseen cases using cross-validation (see below).

For each model, we applied the Least Absolute Shrinkage and Selection Operator (LASSO), which adds the sum of absolute coefficient values to the binomial deviance within the regression equation (Tibshirani, 1996). Our decision to utilize the LASSO penalty was informed by the fact that, unlike some other regularization approaches (such as ridge), coefficients can be reduced to exactly zero, allowing for variable selection. By excluding predictors that do not sufficiently contribute to overall predictive ability, LASSO therefore prioritizes parsimonious models. Where a group of predictor variables are highly correlated, LASSO typically retains only one and shrinks the others to zero; thus, retaining a parsimonious model at a potential cost of a slightly lower prediction accuracy (Hastie, Tibshirani, & Friedman, 2009). However, our predictors were not too highly correlated (*r*=-0.42-0.59).

**Internal validation.** Where wholly independent data are not available, internal validation allows for the evaluation of a model’s predictive ability using ‘unseen’ cases within the current sample. We performed nested 10-fold cross validation to test each model’s internal validity (see Hastie, Tibshirani, & Friedman, 2009). This procedure consists of two nested cross-validation loops (see Figure S3). First, in an outer loop, data are randomly partitioned into ten folds of approximately equal size. Nine of these folds (the blue cubes) are used to estimate or ‘train’ the model, while one fold (the red cube) is retained as ‘test data’ to evaluate model performance. Second, in the inner loop, the training data are used to determine the amount of LASSO regularization applied to model coefficients. This is set by the tuning parameter lambda (*λ*), which can be varied between 0 and 1; as *λ* increases towards 1, coefficient estimates are shrunk closer to zero. In another layer of 10-fold cross-validation, 100 different *λ* values are repeatedly assessed, to determine the value that yields the greatest reduction in prediction error for a tenth fold (yellow cube). Third, the *λ* tuning parameter selected by this inner ‘tuning’ loop is applied to the independent ‘test’ fold originally retained in the outer loop (i.e., the red cube), to determine how well this optimal model performs among ‘unseen’ observations not involved in model development. Finally, this entire process is repeated using a different ‘test’ fold each time, until each of the ten folds defined in the outer loop is used to test model performance once.

**Assessing model performance.** Predictive accuracy was evaluated using the class probabilities generated for each individual when they were treated as a test case (i.e., independent from model development) during nested 10-fold cross-validation. Specifically, we examined: (i) discrimination; (ii) calibration; and (iii) overall precision.

Discrimination refers to the model’s ability to distinguish different classes (i.e., correctly classify victimized children with and without poor functioning). This was examined visually using the receiver operator characteristic (ROC) curve, which plots the model’s true positive rate (i.e., sensitivity) against its false positive rate (i.e., 1 – specificity) across various cut-offs or thresholds for predicted risk (Steyerberg, 2009). The area under the curve (AUC) quantifies the model’s overall discriminative ability. The AUC illustrates the probability that a randomly-selected event case will receive a higher ranking than a randomly-selected non-event. For example, an AUC of 0.70 indicates a 70% probability that a randomly-selected victimized individual who experienced psychosocial disadvantage at age 18 had a higher risk score than a randomly-selected victimized individual who did not. An AUC of 0.5 therefore suggests that a model performs no better than chance, and an AUC of 1 indicates perfect discrimination. For logistic regression models, the following benchmarks have been proposed for discrimination (Hosmer, Lemeshow, & Sturdivant, 2013): <0.7 = poor, 0.7–0.8 = acceptable, 0.8–0.9 = excellent, and 0.9–1.0 = outstanding.

Calibration captures the level of agreement between predictions and observed outcomes. This is assessed visually by plotting predicted probabilities against observed outcomes, where a 45° line indicates perfect calibration. Predictions are systematically higher than actual observed values where the intercept of this plot (known as ‘calibration-in-the large’) is >0, and lower where it is <0. Meanwhile, a calibration slope <1 suggests over-fitting, or that the model is overly sensitive to the specific data being used and may not maintain its current predictive ability when applied to new data. Conversely, a slope >1 suggests under-fitting. A statistical test of unreliability (*U*), based on a chi-square test with two degrees of freedom, was used to determine whether the intercept and slope of each calibration plot was significantly different from the perfect diagonal (i.e., an intercept of 0 and slope of 1; Fenlon, O’Grady, Doherty, & Dunnion, 2018).

Finally, overall model performance was assessed using the Brier score, which represents the mean squared difference between predicted probabilities and actual binary outcomes, capturing aspects of both discrimination and calibration (Brier, 1950). Where the incidence of the outcome is 50%, a Brier score of 0 indicates a perfect model, while a score of 0.25 reflects a non-informative model. As the Brier score will vary depending on the incidence of the outcome, we scaled Brier scores by their maximum possible scores under a non-informative model, where Brier_scaled_ = 1 – Brier / Brier_max_ (Steyerberg, 2009). This scaled Brier score, ranging from 0% (non-informative) to 100% (perfect), has similar interpretation as that of Pearson’s *R*^2^ statistic for continuous outcomes (Hu, Palta, & Shao, 2006). In this way, Brier_scaled_ corresponds to the proportion of the mean squared difference between predicted and observed values associated with a non-informative model that is accounted for by the current model.

**Description of Sensitivity Analyses**

**Regularization penalty.** LASSO regularization, as utilized in our analyses, performs variable selection, particularly when data are highly correlated, to maximize predictive performance in unseen cases using a more parsimonious set of predictors. Accordingly, we sought to assess whether LASSO regularization may have excluded predictors from our models at the expense of predictive accuracy. Within *glmnet*, the tuning parameter alpha (α), ranging from 0–1, determines the relative weight given to one of two penalty terms that can be applied to the prediction error within the regression model (Friedman et al., 2010). The LASSO penalty (i.e., sum of *absolute* coefficients) is denoted by an α of 1 whereas an α of 0 represents the Ridge penalty, which adds the sum of *residual* coefficients to the model’s error term. Ridge regularization does not perform variable selection, but can be better-suited to multicollinear data (James, Witten, Hastie, & Tibshirani, 2013). To test whether a model with the potential to retain more predictors compared to LASSO would show better predictive performance, we re-ran the nested 10-fold cross-validation for each model using ‘elastic net’, a hybrid approach that balances both penalty terms (Zou & Hastie, 2005). Specifically, we set an α of 0.5 as a compromise between Ridge and LASSO penalties that, in turn, de-emphasized the variable selection prioritized by full LASSO regularization (i.e., α = 1).

Tables S6 and S7 display the unstandardized coefficients and internal validation model performance statistics for the elastic net models predicting psychosocial disadvantage and economic disadvantage. The elastic net model predicting psychosocial disadvantage was no different from the LASSO in terms of the variables retained, their coefficient weights or internal validation model performance statistics. The elastic net model predicting economic disadvantage was less parsimonious than the equivalent LASSO model, retaining two additional variables (adult involvement and social cohesion). However, internal validation showed this model did not perform any better than the LASSO. Applying a less restrictive regularization approach, therefore, did not improve model performance for either outcome.

**Non-independence of twins.** We sought to examine whether the presence of non-independent observations within our twin sample may have biased our measures of model performance. Specifically, higher levels of similarity in the risk profiles of twins (compared to singletons) may have inflated predictive accuracy if, during internal validation, the model was ‘trained’ on one twin and ‘tested’ on the other. To address these concerns, we re-ran nested 10-fold cross validation with LASSO regularization in 10 subsamples (*n* = 303–305), each consisting of one twin per twin-pair (where both twins were exposed to childhood victimization, one twin was randomly selected). The results of these sensitivity analyses are presented for each model in Tables S8 and S9. Here, Panel A shows the performance statistics for the full victimization-exposed sample and Panel B shows the performance statistics resulting from each of the 10 single-twin subsamples. For both psychosocial and economic disadvantage, the average prediction performance across these subsamples is similar to the results of the full victimization-exposed sample indicating that our results were not biased by using twins.

**Supplementary Tables**

Table S1

*Exploratory Factor Analysis of Functional Outcome Variables: Eigenvalues and Variance Explained Before Rotation*

| Factor | Eigenvalue | Proportion of variance explained | Cumulative variance explained |
| --- | --- | --- | --- |
| 1 | 2.93 | 0.76 | 0.76 |
| 2 | 1.18 | 0.31 | 1.07 |
| 3 | 0.25 | 0.07 | 1.14 |
| 4 | 0.09 | 0.02 | 1.16 |
| 5 | 0.03 | 0.01 | 1.17 |
| 6 | -0.06 | -0.02 | 1.15 |
| 7 | -0.15 | -0.04 | 1.12 |
| 8 | -0.19 | -0.05 | 1.07 |
| 9 | -0.25 | -0.07 | 1.00 |

*Note*. Two factors were extracted based on eigenvalues >1. The cumulative variance explained exceeds 1 (before returning to = 1) due to Factors 6,7 and 8 having negative eigenvalues.

Table S2

*Confirmatory Factor Analysis of Functional Outcome Variables: Factor Loadings and Variance Explained After Rotation*

| Functional Outcome | Factor 1 | Factor 2 |
| --- | --- | --- |
| Low educational achievement | 0.56 |  |
| NEET | 0.81 |  |
| Parenthood | 0.77 |  |
| Criminal cautions & convictions | 0.55 |  |
| Adolescent poly-victimization | 0.35 | 0.47 |
| Social isolation |  | 0.63 |
| Low life satisfaction |  | 0.83 |
| Loneliness |  | 0.69 |
| Low sleep quality |  | 0.41 |
|  |  |  |
| Variance explained | 0.50 | 0.50 |

*Note*: Loadings <0.30 are not shown. NEET=Not in Education, Employment or Training. Factor 1 conceptualized as ‘economic disadvantage’; Factor 2 conceptualized as ‘psychosocial disadvantage’. Adolescent poly-victimization cross-loaded onto both factors but was included only in Factor 2 where its loading was highest.

Table S3

*Individual, Family, and Community Protective Factors Identified by Meng, Fleury, Xiang, Li, and D’Arcy (2018), Mapped to Variables Measured in the E-Risk Study*

| Protective factors identified by systematic review ^a^ | Variables available in E-Risk measured between ages 5-12 |
| --- | --- |
| **Individual** |  |
| Intelligence | IQ |
| Emotional resources (comprised of: intelligence, positive caregiving, good schools, parental expectations self-esteem, talent, faith, family connectedness financial resources) | IQ, SES, Maternal warmth |
| Personality | Openness to experience, Conscientiousness, Extraversion, Agreeableness, Neuroticism |
| Stable living situation | Number of biological parents in household |
| **Family** |  |
| SES / caregiver education | SES |
| Early family environment / positive family experience / positive parenting / sensitive parenting | Maternal warmth |
| Maternal / parental warmth | Maternal warmth |
| Sibling relationships | Sibling warmth |
| Parental care / years living with biological mother | Number of biological parents in household |
| Maternal depression | Family history of psychopathology |
| Mentorship | Adult involvement |
| Teacher & others caring | Adult involvement |
| Emotional support | Adult involvement |
| **Community** |  |
| Social support | Adult involvement |
| Living in neighborhood with few problems | Neighborhood crime victimization  Social cohesion |
| Peer relationships | Status among peers |

*Note.* ^a^ See Table 3 in Meng et al. (2018) for summary of protective factors. E-Risk=Environmental Risk Longitudinal Twin Study. IQ=intelligence. SES, socioeconomic status.

Table S4

*Classification Measures for the Model Predicting Psychosocial Disadvantage at Age 18 Following Childhood Victimization Across a Range of Dichotomous Risk Score Cut-Offs*

| Risk score cut -off | Predicted prevalence | PPV | NPV | Sensitivity | Specificity |
| --- | --- | --- | --- | --- | --- |
| 40% | 0.98 | 0.62 | 0.63 | 0.99 | 0.03 |
| 50% | 0.83 | 0.66 | 0.58 | 0.89 | 0.26 |
| 60% | 0.52 | 0.73 | 0.50 | 0.61 | 0.64 |
| 70% | 0.25 | 0.74 | 0.42 | 0.30 | 0.83 |
| 80% | 0.10 | 0.76 | 0.40 | 0.12 | 0.94 |
| 90% | 0.02 | 0.82 | 0.38 | 0.03 | 0.99 |

*Note.* PPV = positive predictive value, the proportion of correct model-predicted disadvantage; NPV = negative predictive value, the proportion of correct model-predicted no disadvantage; Sensitivity = model’s ability to correctly identify those with disadvantage; Specificity = model’s ability to correctly identify those without disadvantage. At 60% risk score cut-off the model correctly predicts 61% of individuals who have psychosocial disadvantage at age 18 (sensitivity). It is correct in 73% of its predictions that individuals will develop psychosocial disadvantage at age 18 (PPV) and in 50% of its predictions that they will not (NPV). However, the model has a false positive rate of 36% (1–specificity). Applying the more stringent threshold of 70%, model specificity is improved (from 64% to 83%) but at the expense of sensitivity (which is reduced to 30%). Applying a less stringent threshold improves sensitivity but reduces specificity. Risk score cut-offs begin at 40% due to an absence of cases with risk probabilities <35% in our sample.

Table S5

*Classification Measures for the Model Predicting Economic Disadvantage at Age 18 Following Childhood Victimization Across a Range of Dichotomous Risk Score Cut-Offs*

| Risk score cut -off | Predicted prevalence | PPV | NPV | Sensitivity | Specificity |
| --- | --- | --- | --- | --- | --- |
| 10% | 0.96 | 0.48 | 0.90 | 0.99 | 0.07 |
| 20% | 0.84 | 0.53 | 0.84 | 0.94 | 0.26 |
| 30% | 0.68 | 0.60 | 0.82 | 0.88 | 0.49 |
| 40% | 0.55 | 0.67 | 0.78 | 0.79 | 0.66 |
| 50% | 0.44 | 0.74 | 0.74 | 0.69 | 0.79 |
| 60% | 0.32 | 0.75 | 0.67 | 0.52 | 0.85 |
| 70% | 0.21 | 0.86 | 0.64 | 0.40 | 0.94 |
| 80% | 0.12 | 0.96 | 0.60 | 0.25 | 0.99 |
| 90% | 0.04 | 1.00 | 0.56 | 0.09 | 1.00 |

*Note.* PPV = positive predictive value, the proportion of correct model-predicted disadvantage; NPV = negative predictive value, the proportion of correct model-predicted no disadvantage; Sensitivity = model’s ability to correctly identify those with disadvantage; Specificity = model’s ability to correctly identify those without disadvantage. At 50% risk cut-off the model correctly predicts 69% of individuals who have economic disadvantage at age 18 (sensitivity). It is correct in 74% of its predictions that individuals will develop economic disadvantage at age 18 (PPV) and 74% of its predictions that they will not (NPV). At this threshold, the model has a false positive rate of 21% (1–specificity).

Table S6

*Unstandardized Regression Coefficients for Elastic Net Compared to LASSO Models Predicting Psychosocial Disadvantage (Panel A) and Economic Disadvantage (Panel B)*

|  | Panel A | | Panel B | |
| --- | --- | --- | --- | --- |
|  | Predicting psychosocial disadvantage | | Predicting economic disadvantage | |
|  | LASSO | Elastic Net | LASSO | Elastic Net |
|  | B | B | B | B |
| Intercept | 1.83 | 1.81 | 7.03 | 7.04 |
| Individual: |  |  |  |  |
| IQ | . | . | -0.05 | -0.05 |
| Female sex | 0.05 | 0.05 | -0.17 | -0.18 |
| Openness to experience | . | . | . | . |
| Conscientiousness | . | . | -0.04 | -0.05 |
| Extraversion | . | . | -0.01 | -0.02 |
| Agreeableness | . | . | -0.03 | -0.04 |
| Neuroticism | . | . | -0.002 | -0.02 |
| ADHD | . | . | 0.10 | 0.10 |
| Conduct disorder | 0.14 | 0.12 | . | . |
| Anxiety | 0.05 | 0.05 | . | . |
| Depression | 0.02 | 0.02 | . | . |
| Self-harm/suicide attempt | 0.07 | 0.11 | . | . |
| Psychotic symptoms | . | . | . | . |
| Family: |  |  |  |  |
| Maternal warmth | -0.004 | -0.01 | -0.10 | -0.10 |
| Sibling warmth | -0.02 | -0.02 | -0.03 | -0.03 |
| Adult involvement | -0.06 | -0.06 | . | -0.003 |
| Family history of psychopathology | 0.31 | 0.30 | . | . |
| Number of bio parents in household | . | . | . | . |
| SES | . | . | -0.28 | -0.29 |
| Community: |  |  |  |  |
| Neighborhood crime victimization | 0.08 | 0.07 | . | . |
| Social cohesion | -0.02 | -0.02 | . | -0.01 |
| Status among peers | -0.09 | -0.09 | . | . |
|  |  |  |  |  |
| Deviance explained | 8.8% | 8.7% | 25.0% | 25.2% |

*Note*. ADHD=Attention Deficit Hyperactivity Disorder; LASSO=Least Absolute Shrinkage and Selection Operator; SES=socioeconomic status. Mean minimum lambda for the Elastic net model predicting psychosocial disadvantage=0.04 and for the model predicting economic disadvantage=0.03. Variables with no coefficient reported have been reduced to exactly zero and thus removed from the model.

Table S7

*Performance of the Elastic Net Models Compared to LASSO Models Predicting Psychosocial Disadvantage (Panel A) and Economic Disadvantage (Panel B)*

|  | Panel A | | Panel B | |
| --- | --- | --- | --- | --- |
| Risk prediction performance measure | Predicting psychosocial disadvantage | | Predicting economic disadvantage | |
|  | LASSO Model | Elastic Net Model | LASSO Model | Elastic Net Model |
| Discrimination: |  |  |  |  |
| AUC | 0.65 | 0.66 | 0.80 | 0.80 |
| Calibration: |  |  |  |  |
| Calibration-in-the-large | 0.07 | 0.03 | 0.01 | 0.02 |
| Calibration slope | 0.84 | 0.91 | 1.11 | 1.13 |
| Unreliability *p*-value | .677 | .900 | .570 | .515 |
| Overall: |  |  |  |  |
| Scaled Brier score (%) | 5.7 | 5.9 | 27.8 | 27.5 |

*Note.* AUC=Area Under the Curve*;* LASSO=Least Absolute Shrinkage and Selection Operator.

Table S8

*Twin Sensitivity Analyses: Performance Measures for LASSO Model Predicting Psychosocial Disadvantage*

| Risk prediction performance measure | Panel A | Panel B | | | | | | | | | | |
| --- | --- | --- | --- | --- | --- | --- | --- | --- | --- | --- | --- | --- |
|  | Victimized participants (*n* = 506) | Victimized group, subsamples consisting of one twin per twin pair (*n* = 305) | | | | | | | | | | |
|  |  | 1 | 2 | 3 | 4 | 5 | 6 | 7 | 8 | 9 | 10 | Average |
| Discrimination: |  |  |  |  |  |  |  |  |  |  |  |  |
| AUC | 0.65 | 0.57 | 0.65 | 0.59 | 0.63 | 0.60 | 0.59 | 0.65 | 0.61 | 0.66 | 0.67 | 0.62 |
| Calibration: |  |  |  |  |  |  |  |  |  |  |  |  |
| Calibration-in-the-large | 0.07 | 0.24 | 0.12 | 0.31 | 0.14 | 0.17 | 0.24 | 0.06 | 0.11 | -0.05 | 0.07 | 0.14 |
| Calibration slope | 0.84 | 0.54 | 0.82 | 0.49 | 0.75 | 0.68 | 0.55 | 0.84 | 0.79 | 1.12 | 0.88 | 0.75 |
| Overall: |  |  |  |  |  |  |  |  |  |  |  |  |
| Scaled Brier score (%) | 5.7 | 0.7 | 5.5 | 1.8 | 4.0 | 1.7 | 2.0 | 4.8 | 3.6 | 5.8 | 7.3 | 3.7 |

*Note.* AUC=Area Under the Curve; LASSO=Least Absolute Shrinkage and Selection Operator.

Table S9

*Twin Sensitivity Analyses: Performance Measures for LASSO Model Predicting Economic Disadvantage*

| Risk prediction performance measure | Panel A | Panel B | | | | | | | | | | |
| --- | --- | --- | --- | --- | --- | --- | --- | --- | --- | --- | --- | --- |
|  | Victimized participants (*n* = 503) | Victimized group, subsamples consisting of one twin per twin pair (*n*=302) | | | | | | | | | | |
|  |  | 1 | 2 | 3 | 4 | 5 | 6 | 7 | 8 | 9 | 10 | Average |
| Discrimination: |  |  |  |  |  |  |  |  |  |  |  |  |
| AUC | 0.80 | 0.80 | 0.79 | 0.77 | 0.80 | 0.81 | 0.80 | 0.78 | 0.79 | 0.79 | 0.79 | 0.79 |
| Calibration: |  |  |  |  |  |  |  |  |  |  |  |  |
| Calibration-in-the-large | 0.01 | 0.02 | 0.04 | 0.03 | 0.00 | 0.02 | 0.02 | 0.03 | 0.02 | 0.02 | 0.00 | 0.02 |
| Calibration slope | 1.11 | 1.12 | 1.14 | 1.10 | 1.08 | 1.09 | 1.11 | 1.12 | 1.12 | 1.12 | 1.09 | 1.11 |
| Overall: |  |  |  |  |  |  |  |  |  |  |  |  |
| Scaled Brier score (%) | 27.8 | 25.8 | 25.1 | 23.1 | 26.7 | 29.1 | 27.6 | 24.7 | 25.6 | 24.4 | 26.2 | 25.8 |

*Note.* AUC=Area Under the Curve; LASSO=Least Absolute Shrinkage and Selection Operator.

**Supplementary Figures**


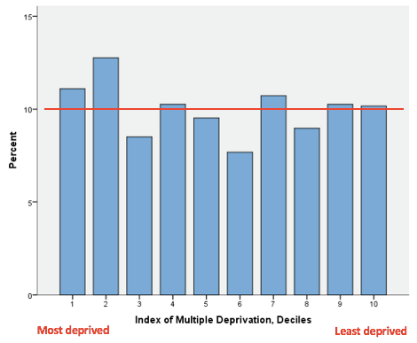


***Figure S1.*** Approximately 10% of E-Risk families’ fall in each of the 10% decile categories of the Index of Multiple Deprivation (IMD) for the UK (see page 1 of Supplementary Material for description).

A

B

C

D

*Figure S2*. A regression line that is fitted very closely to the data (Panel A) may not generalize very well to new data (brown dots in Panel B) – this is the problem of ‘overfitting’. LASSO regularized regression attempts to minimize this problem by fitting a regression line that is a slightly worse fit to the data (Panel C) but that will generalize better to new data (brown dots in Panel D). It does this by introducing bias into the model that shrinks the regression coefficients – this ensures the regression model does not fit the data it is developed in too closely and will therefore be better at making predictions in new data.

***
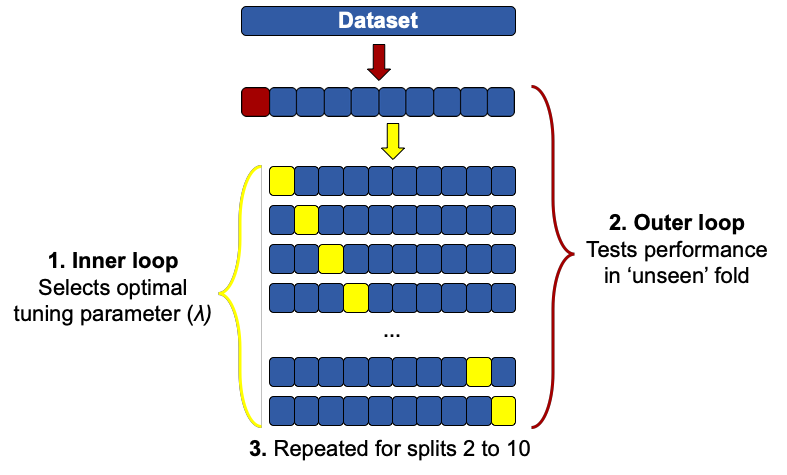
***

*Figure S3*. Illustrated example of nested 10-fold cross-validation analysis. See page 18 of Supplementary Material for description.

**References**

Achenbach, T. M. (1991a). *Manual for the Child Behavior Checklist/4-18 and 1991 Profile*. Burlington, VT: University of Vermont Department of Psychiatry.

Achenbach, T. M. (1991b). *Manual for the Teacher’s Report Form and 1991 Profile*. Burlington, VT: University of Vermont Department of Psychiatry.

Arseneault, L., Walsh, E., Trzesniewski, K., Newcombe, R., Caspi, A., & Moffitt, T. E. (2006). Bullying victimization uniquely contributes to adjustment problems in young children: A nationally representative cohort study. *Pediatrics, 118,* 130- 138.

Asher, S. R., & Wheeler, V. A. (1985). Children’s loneliness: a comparison of rejected and neglected peer status. *Journal of Consulting and Clinical Psychology, 53,* 500-505.

Bowes, L., Maughan, B., Ball, H., Shakoor, S., Ouellet-Morin, I., Caspi, A., Moffitt, T.E., & Arseneault, L. (2013). Chronic bullying victimization across school transitions: the role of genetic and environmental influences. *Developmental Psychopathology, 25,* 333-346.

Bradley, R., & Caldwell, B. (1977). Home observation for measurement of the environment: a validation study of screening efficiency. *American Journal of Mental Deficiency, 81,* 417-420.

Brier, G. W. (1950). Verification of forecasts expressed in terms of probability. *Monthly Weather Review, 78*, 1-3.

Buysse, D. J., Reynolds, C. F., Monk, T. H., Berman, S. R., & Kupfer, D. J. (1989). The Pittsburgh Sleep Quality Index: a new instrument for psychiatric practice and research. *Psychiatry Research, 28,* 193–213.

Caspi, A., Moffitt, T. E., Thornton, A., Freedman, D., Amell, J. W., Harrington, H., Smeijers, J., & Silva, P. A. (1996). The life history calendar: a research and clinical assessment method for collecting retrospective event-history data. *International Journal of Methods in Psychiatric Research, 6,* 101-114.

Danese, A., Moffitt, T.E., Arseneault, L., Bleiberg, B.A., Dinardo, P.B., Gandelman, S.B., Houts, R., Ambler, A., Fisher, H.L., & Poulton, R. (2017). The origins of cognitive deficits in victimized children: implications for neuroscientists and clinicians. *American Journal of Psychiatry, 174,* 349-361.

Diener, E., Emmons, R. A., Larsen, R. J., Griffin, S. (1985). The Satisfaction with Life Scale. *Journal of Personality Assessment, 49*, 71-75.

Dodge, K.A., Bates, J.E., & Pettit, G. S. (1990). Mechanisms in the cycle of violence. *Science, 250,* 1678-1683.

Fenlon, C., O’Grady, L., Doherty, M. L., & Dunnion, J. (2018). A discussion of calibration techniques for evaluating binary and categorical predictive models. *Preventive Veterinary Medicine, 149*, 107-114.

Finkehor, D., Hamby, S. L., Turner, H. A., & Ormrod, R. K. (2011). The Juvenile Victimization Questionnaire: 2nd Revision (JVQ-R2) Durham, NH: Crimes Against Children Research Center.

Fisher, H. L., Caspi, A., Moffitt, T. E., Wertz, J., Gray, R., Newbury, J., … & Arseneault, L. (2015). Measuring adolescents’ exposure to victimization: The Environmental Risk (E-Risk) longitudinal twin study. *Development and Psychopathology, 27,* 1399–1416.

Fisher, H. L., Moffitt, T. E., Houts, R. M., Belsky, D. W., Arseneault, L., & Caspi, A. (2012). Bullying victimisation and risk of self harm in early adolescence: Longitudinal cohort study. *British Medical Journal, 344*(e2683).

Fletcher, J. M. (2010). Adolescent depression and educational attainment: results using sibling fixed effects. *Health Economics, 19*, 855-871.

Friedman, J., Hastie, T., & Tibshirani, R. (2010). Regularization paths for generalized linear models via coordinate descent. *Journal of Statistical Software, 33*, 1-22.

Goldman-Mellor, S., Caspi, A., Arseneault, L., Ajala, N., Ambler, A., Danese, A., … Moffitt, T. E. (2016). Committed to work but vulnerable: Self-perceptions and mental health in NEET 18-year olds from a contemporary British cohort. *Journal of Child Psychology and Psychiatry, 57,* 196-203.

Hamby, S., Finkelhor, D., Ormrod, D., & Turner, H. (2004). The comprehensive JVQ administration and scoring manual. Durham, NH: University of New Hampshire, Crimes Against Children Research Centre.

Hastie, T., Tibshirani, R., & Friedman, J. (2009). *The elements of statistical learning: Data mining, inference, and prediction* (2nd ed.). New York, NY: Springer.

Hosmer, D. W., Lemeshow, S., & Sturdivant, R. X. (2013). *Applied logistic regression* (3rd ed.). New York: John Wiley & Sons

Hu, B., Palta, M., & Shao, J. (2006). Properties of R(2) statistics for logistic regression. *Statistics in Medicine, 25*, 1383-1395.

Hughes, M. E., Waite, L. J., Hawkley, L. C., & Cacioppo, J. T. (2004) A short scale for measuring loneliness in large surveys: results from two population-based studies. *Research on Aging, 26,* 655–672.Jaffee, S. R., Caspi, A., Moffitt, T. E., Polo-Tomas, M., & Taylor, A. (2007). Individual, family, and neighborhood factors distinguish resilient from non-resilient maltreated children: a cumulative stressors model. *Child Abuse & Neglect, 31,* 231–253.

Jaffee, S.R., Caspi, A., Moffitt, T.E., & Taylor, A. (2004). Physical maltreatment victim to antisocial child: evidence of an environmentally mediated process. *Journal of Abnormal Psychology, 113,* 44-55.

James, G., Witten, D., Hastie, T., & Tibshirani, R. (2013). *An introduction to statistical learning*. New York: Springer.

John, O. P., & Srivastava, S. (1999). *The Big Five trait taxonomy: History, measurement, and theoretical perspectives.* In L. A. Pervin & O. P. Joh (Eds.), Handbook of personality: Theory and research (2^nd^ ed) pp. 102-138. New York: Guildford Press.

Kovacs, M. (1992). *Children's Depression Inventory (CDI) Manual*. Toronto, ON: Multi-Health Systems.

Lansford, J. E., Dodge, K. A., Pettit, G. S., Bates, J. E., Crozier, J., Kaplow, J. (2002). A 12-year prospective study of the long-term effects of early child physical maltreatment on psychological, behavioral, and academic problems in adolescence. *Archives of Pediatrics and Adolescent Medicine, 156*, 824-830.

Magaña, A. B., Goldstein, J. M., Karno, M., Miklowitz, D. J., Jenkins, J., & Falloon. I. R. (1986). A brief method for assessing expressed emotion in relatives of psychiatric patients. *Psychiatry Research, 17,* 203–212.

March, J. S., Parker, J. D., Sullivan, K., Stallings, P., & Conners. C. K., (1997). The Multidimensional Anxiety Scale for Children (MASC): Factor structure, reliability, and validity. *Journal of the American Academy of Child & Adolescent Psychiatry, 36,* 554–565.

Meng, X., Fleury, M. J., Xiang, Y. T., Li, M., & D’Arcy, C. (2018). Resilience and protective factors among people with a history of child maltreatment: a systematic review. *Social Psychiatry and Psychiatric Epidemiology*, 53, 453-475.

Milne, B. J., Caspi, A., Crump, R.., Poulton, R., Rutter, M., Sears, M. R., & Moffitt, T. E. (2009). The validity of the family history screen for assessing family history of mental disorders. *American Journal of Medical Genetics Part B: Neuropsychiatric Genetics, 150,* 41–49.

Moffitt, T. E., Caspi, A., Krueger, R. F., Lynn, M., Gayla, M., Phil, A. S., & Ros, S. (1997). Do partners agree about abuse in their relationship? A psychometric evaluation of interpartner agreement. *Psychological Assessment, 9*, 47-56.

Office for National Statistics. (2013). *UK Estimate of Young People Not in Education, Employment or Training*. London, UK.

Office for National Statistics. (2014). *Young People Not in Education, Employment or Training (NEET).* London, UK.

Office for National Statistics. (2016). *Childbearing for women born in different years.* London, UK.

Polanczyk, G., Moffitt, T. E., Arseneault, L., Cannon, M., Ambler, A., Keefe, R. S. E., Houts, R., Odgers, C. L., & Caspi, A. (2010). Etiological and clinical features of childhood psychotic symptoms: results from a birth cohort. *Archives of General Psychiatry, 67,* 328–338.

Russell, D. W. (1996). UCLA Loneliness Scale (Version 3): reliability, validity, and factor structure. *Journal of Personality Assessment, 66,* 20–40.

Sampson, R. J., Raudenbush, S. W., & Earls, F. (1997). Neighborhoods and violent crime: a multilevel study of collective efficacy. *Science, 277,* 918–924.

Sattler, J. M. (2008). *Assessment of children: Cognitive foundations* (5th ed.). San Diego, CA: JM Sattler.

Shakoor, S., Jaffee, S., Andreou, P., Bowes, L., Ambler, A. P., Caspi, A., Moffitt, T. E., & Arseneault, L. (2011). Mothers and children as informants of bullying victimization: Results from an epidemiological cohort of children. *Journal of Abnormal Child Psychology, 39,* 379–387.

Straus, M. A. (1990). *Measuring intrafamily conflict and violence: The Conflict Tactics (CT) scales.* In: Straus MA, Gelles RG, editors. Physical violence in American families: risk factors and adaptations to violence in 8,145 families. New Brunswick, NJ: Transaction Press, pp. 403–424.

Steyerberg, E. W. (2009). *Clinical prediction models: A practical approach to development, validation, and updating*. New York: Springer.

Tibshirani, R. (1996). Regression shrinkage and selection via the Lasso. *Journal of the Royal Statistical Society. Series B (Methodological), 58*, 267-288.

Trzesniewski, K. H., Donnellan, M. B., Moffitt, T. E., Robins, R. W., Poulton, R., & Caspi, A. (2006). Low self-esteem during adolescence predicts poor health, criminal behavior, and limited economic prospects during adulthood. *Developmental Psychology,* *42*, 381-390.

Wertz, J., Agnew-Blais, J., Caspi, A., Danese, A., Fisher, H. L., Goldman-Mellor, S., ... Arseneault, L. (2018). From childhood conduct problems to poor functioning at age 18 years: Examining explanations in a longitudinal cohort study. *Journal of the American Academy of Child & Adolescent Psychiatry, 57,* 54-60.

Wechsler, D. (1974). *Manual for the Wechsler Intelligence Scale for Children—Revised*. New York, NY: Psychological Corporation.

Woodward, L. J., & Fergusson, D. M. (2001). Life course outcomes of young people with anxiety disorders in adolescence. *Journal of the American Academy of Child & Adolescent Psychiatry, 40*, 1086-1093.

Zimet, G. D., Dahlem, N. W., Zimet, S. G., & Farley, G. K. (1988). The Multidimensional Scale of Perceived Social Support. *Journal of Personality Assessment, 52*, 30-41.

Zou, H., & Hastie, T. (2005). Regularization and variable selection via the elastic net. *Journal of the Royal Statistical Society: Series B (Statistical Methodology), 67*, 301-320.
